# Lack of striatal-enriched protein tyrosine phosphatase affected the serotonin system, behavior, and brain morphology in mice

**DOI:** 10.3389/fpsyt.2025.1730197

**Published:** 2026-01-14

**Authors:** Vitalii Moskaliuk, Polina Komleva, Nikita Khotskin, Alla Arefieva, Oleg Shevelev, Alexey Korablev, Irina Serova, Nariman Battulin, Alexander Kulikov, Vladimir Naumenko, Darya Bazovkina, Elizabeth Kulikova

**Affiliations:** 1 Institute of Cytology and Genetics, Siberian Branch of Russian Academy of Sciences, Novosibirsk, Russia; 2 Federal State Autonomous Educational Institution for Higher Education National Research Tomsk Polytechnic University, International Research Center of Piezo and Magnetoelectric Materials, Tomsk, Russia; 3 State Research Center of Virology and Biotechnology “Vector”, Federal Service for Surveillance on Consumer Rights Protection and Human Well-Being (FBRI SRC VB “Vector”, Rospotrebnadzor), Koltsovo, Russia

**Keywords:** behavior, brain, mental disorders, mice, PTPN5, serotonin system, striatal-enriched protein tyrosine phosphatase STEP

## Abstract

**Introduction:**

Mental disorders are a severe problem of modern society. Significant in these conditions are the striatal-enriched protein tyrosine phosphatase (STEP) (*Ptpn5* gene) and the serotonergic system. Nevertheless, the association between them is poorly studied. The aim of this research was to investigate the effects of *Ptpn5* gene knockout on behavior and the serotonin system in mice.

**Methods:**

Utilizing the CRISPR/Cas9 system, we cleaved the PTP-domain-encoding sequence from the *Ptpn5* gene of C57BL/6 mice. The resulting strain (*Ptpn5* KO) demonstrated STEP protein absence and ERK1/2 hyperphosphorylation (STEP substrate) in the brain. We performed behavioral phenotyping, structural magnetic resonance imaging (MRI) and biomolecular screening of the serotonergic system.

**Results:**

*Ptpn5* KO mice resembled the wild type in locomotor activity, motor function, and social behavior. They were overactive during dark hours and showed reduced anxiety-related behavior, elevated grooming activity, and an increased pre-pulse inhibition index. Mutant mice performed poorly in the water-related tests. They demonstrated higher immobility time in the forced swim test but not in the analogous dry tail suspension test, and experienced difficulty finding the platform in the Morris water maze but did not fail the novel object recognition test or the operant wall task. Therefore, the observed differences may be a reaction to environmental stress rather than depressive-like behavior or learning deficiency. The *Ptpn5* KO strain had a bigger cortex and striatum but a smaller midbrain and cerebellum. Serotonin and its metabolite content was lower in the frontal cortex and higher in the midbrain of *Ptpn5* KO mice. A lack of STEP elevated TPH2 protein level in the hippocampus and reduced *Htr1a* and *Htr7* mRNA expression in the midbrain and hippocampus, respectively.

**Discussion:**

The data obtained in this study indicate a significant role of STEP in the regulation of behavior and brain architecture, and highlight the connection between STEP and the 5-HT system.

## Introduction

Mental illnesses are the leading cause of disability and life quality deterioration globally. In the last decade, particular attention has been paid to the striatal-enriched protein tyrosine phosphatase (STEP), encoded by the *Ptpn5* gene (1). It has been shown that STEP is involved in the pathogenesis of numerous neurodegenerative and psychiatric disorders, as well as in response to stress, brain damage, and aging (for a detailed review, see 2). This derives from STEP’s crucial role in the maintenance of a fragile balance in the nervous cell. STEP dephosphorylates a number of intracellular signaling kinases, such as ERK1/2, Fyn, Pyk2, and p38 (3–6) that are involved in synaptic regulation, neuroplasticity, and cell survival/death determination cascades, as well as glutamate receptor subunits (7, 8) that play a key role in long-term potentiation and depression.

Another prominent player in the pathogenesis of mental disorders is the brain serotonin (5-HT) system. Its dysregulation is considered one of the causes of depression and anxiety disorders (9), obsessive–compulsive disorder (10), and post-traumatic stress disorder (11). The 5-HT system is involved in the control of a great number of physiological processes as well as behavioral and psychological traits. It has been implicated in the coordination of the sleep cycle, sexual behavior, motor control, appetite, and digestion as well as in mood regulation, aggressive behavior, memory, and cognition (for review, see 12). This multifaceted role of 5-HT in the nervous system is in part established by a wide variety of its receptors (13). 5-HT is synthesized from amino acid tryptophan in two stages; the rate-limiting stage is catalyzed in the CNS by the tryptophan hydroxylase 2 (TPH2) enzyme (14). In the synaptic cleft, 5-HT acts via pre- and postsynaptic receptors and is subsequently returned to the 5-HT neuron by the serotonin transporter (5-HTT) (15) where it is metabolized by monoamine oxidase A (MAOA) to 5-hydroxyindolacetic acid (5-HIAA) (16).

Although both STEP and the 5-HT system are involved in a number of mutual processes and diseases, there is a lack of data considering the STEP and 5-HT interplay, despite some indirect evidence of this link. The STEP inhibitor TC-2153 has been shown to act upon several components of the 5-HT system. TC-2153 administration increases levels of 5-HT and its metabolite in the hypothalamus of mice (17), decreases 5-HT_2A_ receptor activity and protein level (18), differentially affects mRNA levels of several 5-HT receptors (19, 20), and alters the enzymatic activity and expression of the main 5-HT system enzymes TPH2 and MAOA (21). Conversely, pharmacological influence on the 5-HT system induces changes in STEP level and activity: acute administration of selective 5-HT reuptake inhibitors increases STEP activity in the brain of zebrafish (22), whereas TPH2 and MAOA inhibitors decrease mouse *Ptpn5* gene expression (23) and STEP activity in zebrafish (24).

In addition, experiments with *Ptpn5* gene knockout and TC-2153 suggest that STEP plays a significant role in the regulation of spatial learning, dominant behavior (25), prepulse inhibition (26), social memory, stress coping, exploratory activity (27), anxiety (27, 28), depression (18, 29), and aggression (28). There is evidence of 5-HT system involvement in all these processes (9, 12). While *Ptpn5* KO mice have been extensively utilized to study the role of STEP in the cellular processes (5–8, 30) and nervous system disorders (7, 31–36), there is a substantial void in the characterization of their 5-HT system, particularly in relation to the observed behavioral phenotype.

To more consistently investigate the crosstalk between STEP and the 5-HT system, we created a mouse strain expressing an inactive STEP protein that lacks the phosphatase domain (*Ptpn5* KO mice). In this work, we evaluated the effect of *Ptpn5* knockout on behavior, brain region volume, and the 5-HT system in mice.

## Materials and methods

### Generation of Ptpn5-KO line

**Figure 1:**
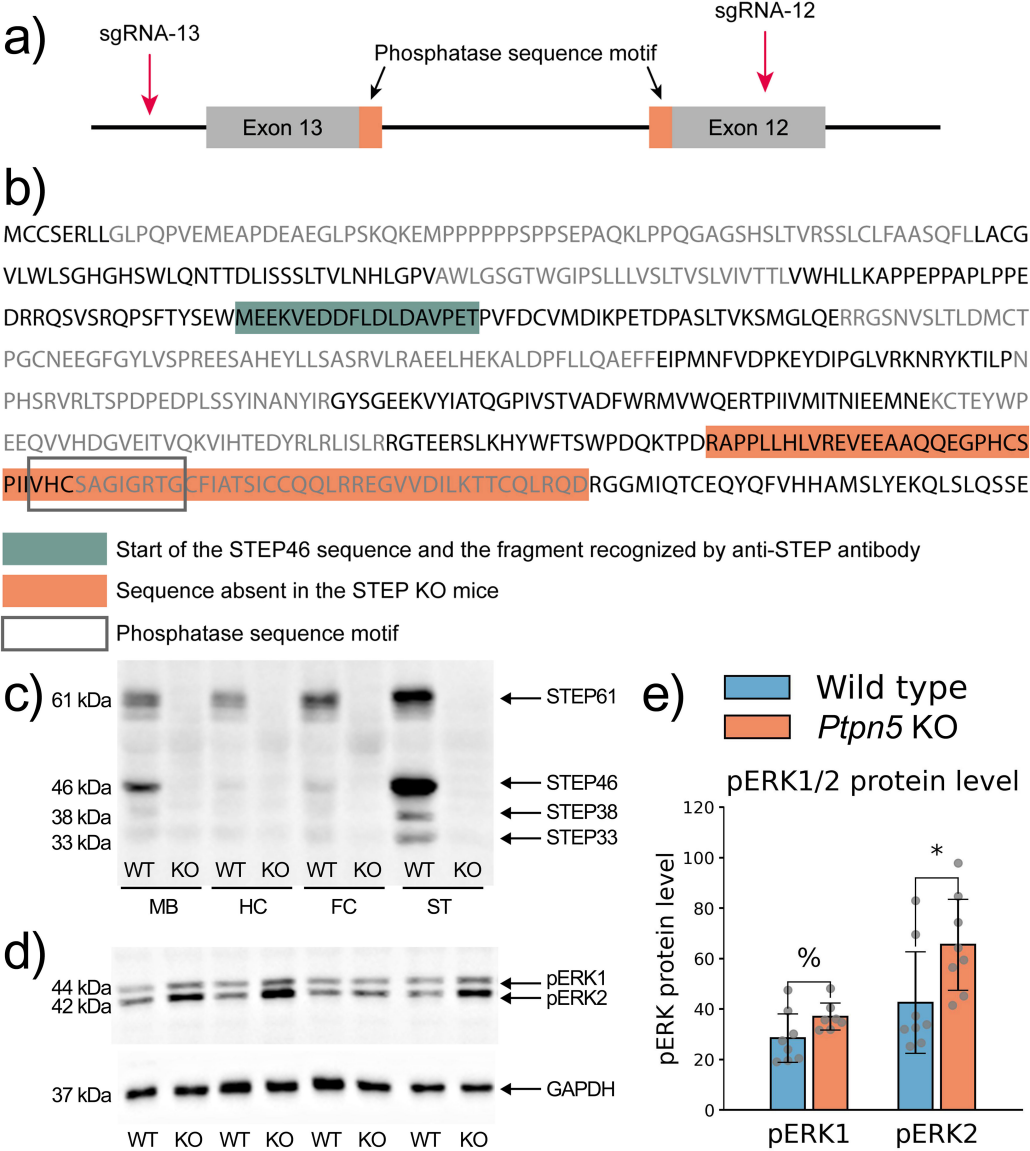
**(A)** Scheme of the CRISPR/Cas9 system applied to mouse *Ptpn5* gene; **(B)** amino acid sequence of mouse STEP protein encoded by the *Ptpn5* gene. Dark and light gray lettering indicates the exonal structure. Green highlighted sequence is the translation start of the STEP46 isoform and the antigen sequence for the anti-STEP antibody (sc-23892, Santa Cruz Biotechnology, USA). The orange highlighted sequence is absent in the *Ptpn5* KO mice. Rectangle shows the phosphatase sequence motif; **(C–E)** protein quantification by Western blot analysis. **(C)** STEP protein is absent in the frontal cortex (FC), hippocampus (HC), striatum (ST), and midbrain (MB) of Ptpn5 KO mice. **(D, E)** STEP substrates ERK1/2 kinases exhibit elevated phosphorylation levels in the striatum of *Ptpn5* KO mice. pERK1/2 protein levels were normalized to the amount of GAPDH protein. *p < 0.05, ^%^*p* = 0.07 compared to the wild type (seven to eight animals per group). Groups were compared with *t*-test for independent samples.

STEP is a protein tyrosine phosphatase; its catalytic domain (PTP-sequence) is located at amino acids positions 470–480 and is encoded in exons 12 and 13. To generate a mouse strain with a functional STEP knockout, we removed the PTP sequence from the *Ptpn5* gene using the CRISPR/Cas9 system and applied the previously described approach (37, 38) (**Figure 1A**).

### CRISPR system design and preparation of microinjections components

Single guide RNAs (sgRNAs) were designed with the Benchling online tool (https://benchling.com) (**Table 1**) using the scoring method described previously (39). Genotyping primers were created using the Primer Blast tool (https://www.ncbi.nlm.nih.gov/tools/primer-blast) (**Table 1**). The sgRNAs were synthetized with the HiScribe™ T7 High Yield RNA Synthesis kit (NEB, Ipswich, MA, USA) and were purified with the RNA Clean & Concentrator-25 kit (Zymo Research, Irvine, CA, USA).

**Table 1:** Sequences utilized in the CRISPR system and genotyping primers.

Name	Sequence
sgRNA-12	5′-TGACCAGAAGACCCCCGACCGGG-3′
sgRNA-13	5′-CATGATGTGAGCGTGAATCGAGG-3′
Ptpn5-F	5′-GTGGGCAGACAGAGCATAGT-3′
Ptpn5-R	5′-CCTCTGCCCCTTCCTTTCAG-3′
ssODN	5′-TGGTTTTTCAGTTCCCTAGCCACCTTACACACTATGTCTGGTTTGTCAGCTATCCCTCGACGGGGGTCTTCTGGTCAGGCCAGGATGTGAACCAGTAATGCTTCAAGCTTCGCTCTTCAGTCCCTCT-3′

### Generation of *Ptpn5* knockout mice

Mice were bred at the SPF animal facility of the Institute of Cytology and Genetics (Novosibirsk, Russia). After weaning, mice of the same sex were kept in groups of four to five animals per cage (Optimice, Animal Care Systems, Centennial, CO, USA). Caging conditions were kept at 24 ± 2°C, 45%–50% humidity, and a 14:10 dark–light cycle (lights on at 01:00; lights off at 15:00). Food and water were provided *ad libitum*. The food and litter were autoclaved at 121°C before use. Deionized water (produced in a Millipore device) was added with a Severyanka mineral supplement (Eko-proekt, St. Petersburg, Russia).

*In vitro* fertilization was performed as described earlier (40). Oocytes and spermatozoa were taken from C57BL/6 females (4–6 weeks old) and C57BL/6 males (3 months old), respectively. Oocytes were microinjected with a solution containing 100 ng/μL ssODN (**Table 1**), 25 ng/μL sgRNA, and an equimolar concentration of Alt-R HiFi Cas9 Nuclease V3 (IDT, Coralville, IA, USA) into the cytoplasm. After the microinjection, embryos were cultivated overnight, and 2-cell stage embryos were transferred into the oviducts of pseudopregnant mothers (females of the CD-1 strain) (41).

Ptpn5-F and Ptpn5-R primers (**Table 1**) were utilized in the genotyping of the offspring to detect the 347-bp deletion (chr 7:46728942–46729288, GRCm39/mm39). One founder from six founders with the expected deletion was selected for breeding and generating a *Ptpn5* knockout strain. The obtained mouse strain was named C57BL/6-Ptpn5_KO (listed in the SPF animal facility of the Institute of Cytology and Genetics catalogue by the name “C57BL/6-Ptpn5-KO-ICG”).

### Animals and procedures

Experiments were carried out on adult 2-month-old male mice of the C57BL/6-Ptpn5_KO (*Ptpn5* knockout mice) and C57BL/6 (wild type) inbred strains, 24 ± 1 g of weight. In this study, only male mice were used to exclude the influence of the hormonal background on the behavioral tests. Mice were kept in SPF-state conditions as described above. Two days before the tests, the animals were isolated to reduce group effects. Behavioral tests were held between 15:00 and 18:00 in the dark. All procedures were conducted in the strict accordance with the recommendations of the Directive 2010/63/EU of the European Parliament and of the Council of 22 September 2010 on the protection of animals used for scientific purposes and was approved by the Committee on the Ethics of Animal Experiments of the Russian National Center of Genetic Resources of Laboratory Animals of Institute of Cytology and Genetics of Russian Academy of Sciences (protocol No. 96 of 25 October 2021). All sample sizes were chosen as a compromise between the requirements of statistical correctness and minimization of the number of experimental animals.

### Experimental design

Experiment 1. The mice were tested in the marble burying test (MBT) and, on the next day, in the novel object recognition (NOR) test for three subsequent days. Then, the behavior was analyzed for the three subsequent days in the social-interaction test, elevated plus-maze (EPM) test, and three-chambered social approach test. After 1 month of rest, the startle test was performed.

Experiment 2. The behavior of mice was evaluated in the open field (OF) test, in the forced swim test (FST) on the next day, and in the tail suspension test (TST) after 1 day of rest.

Experiment 3. The mice’s performance was assessed in the OF, in the Morris water maze (MWM) on the next 5 days, and in the rotarod test after 2 days of rest. After 1 day of rest, the functional activity of the 5-HT_1A_ receptor was measured; the next day, the functional activity of the 5-HT_2A_ receptor was studied; and after 2 days of rest, the functional activity of the 5-HT_7_ receptor was evaluated.

Experiment 4. Home cage activity was registered and the operant wall paradigm was carried out over 3 days. Two days later, the animals were euthanized by carbon dioxide asphyxiation followed by decapitation. The frontal cortex, hippocampus, striatum, and midbrain were rapidly dissected, frozen in liquid nitrogen, and stored at −80°C for further assay of monoamine content, TPH2 activity, proteins, and gene expression. The selected brain regions exhibit the most abundant expression of *Ptpn5* RNA (1) and are involved in the 5-HT system regulated processes: mood regulation, decision-making, and depressive and anxiety-related behavior; midbrain is home for 5-HT nuclei.

Experiment 5. The brain morphology was studied with magnetic resonance imaging (MRI); total volumes of the whole brain, cortex, striatum, interbrain, hippocampus, pituitary, midbrain, and cerebellum were determined.

### Behavioral testing

#### Open field test

The OF test was carried out to assess the locomotor and exploratory activity. The test took place at the circular arena, 60 cm in diameter (**Supplementary Figures S1A, B**), as described earlier (42). Movement of the mouse was automatically traced for 5 min with a digital camera. The total distance traveled (m) and time (%) spent in the center of the arena were automatically evaluated by the EthoStudio software (43). The number of vertical postures (rearing) and the number of grooming episodes were marked by experienced rater blinded to the experimental group assignment. After each test, the arena was cleaned with wet (H_2_O_2_) and dry napkins.

#### Forced swim test

In the FST and TST, mice were tested for depressive-like behavior. The FST was performed in a clear, cylindrical glass reservoir (*h* = 30 cm, *d* = 15 cm) half filled with water (*T* = 25°С) and illuminated from beneath. A mouse was carefully placed into the water for 6 min. The first 2 min of the test are adaptive and were not analyzed. For the latter 4 min, the total immobility time was evaluated by an experienced rater, and depressive-like behavior was determined in correspondence to this parameter.

#### Tail suspension test

A mouse was fixated by the tail with an adhesive tape to a horizontal bar placed 30 cm above the table surface. During the 6 min of the test, immobility episodes, during which a mouse was passive and hung motionless, were recorded by the researcher. Depressive-like behavior was evaluated by the total immobility episodes duration.

#### Marble burying test

The MBT serves to evaluate the stereotypical behavior associated with obsessive ideas and actions as well as anxiety-related behavior (44). Eighteen identical brightly colored glass marbles (*d* = 1 cm) were evenly distributed across the clean cage (Optimice, Animal Care Systems, Inc., USA) filled with sawdust layer 4 cm deep. A mouse was placed alone in the cage with marbles for 30 min. Afterwards, the mouse was removed and the number of buried marbles was counted. A marble was considered buried if the sawdust covered over 2/3 of its surface.

#### Novel object test

The NOR test is utilized to test for the memory performance of mice. We followed the previously described procedure (45). The time spent near the objects was registered automatically. The novelty index was calculated as follows: time spent with new object (No)/[time spent with new object (No) + time spent with familiar object (Fo)] × 100%.

#### Social interaction test

The test followed the “resident–intruder” paradigm. A juvenile Balb/c male (4 weeks old) was introduced to the home cage of the tested male. During 10 min, social interactions were registered with the EthoStudio software. Social behavior was evaluated as the total duration of social contacts (intruder’s head and body sniffing).

#### Elevated plus-maze test

The test was carried out in the apparatus made of gray plastic consisting of four arms connected perpendicularly (closed and open, 30 × 6 cm each) with a central area (6 × 6 cm) (**Supplementary Figures S2A, B**). The closed arms were bordered with 20-cm-high walls. The device was elevated by 60 cm above the floor and dimly illuminated with diffuse lighting (100 lx) of a halogen lamp (25 W) placed under the device. The animal’s movements were automatically traced with a 3D sensor Kinect 1 connected to the PC through a USB-2 port. During the 5 min of testing, the sensor automatically detected the animal’s movement in the open as well as the closed arms. The total path (m), time (%) spent in the center, and open and closed arms were automatically computed by the EthoStudio software. Stretch poses and head dips from the open arms were counted by an experienced rater. The arena was cleaned with wet (H_2_O_2_) and dry napkins after each test.

#### Three-chambered social approach test

To evaluate a mouse’s sociability, a three-chambered social approach test was performed following the protocol described elsewhere (46). During the time of testing, the researcher left the room. Time spent in each chamber was recorded. Movement tracking was performed with Kinect 3D sensor (Microsoft Corporation, USA) connected to the PC through the USB-2 port (47). Time spent in each chamber was evaluated with the EthoStudio software (43). The preference index was calculated as a time (%) spent in the chamber with the “guest” mouse relative to total time. Between tests, the device and all used materials were cleaned with wet (H_2_O_2_) and dry napkins.

#### Rotarod test

To assess balance and motor coordination, mice were tested on a rotor-rod device (San Diego Instruments, USA). A mouse was placed on the rod with a rotation frequency gradually increasing from 5 to 40 rpm within 5 min. The latency time (s) and rotation frequency (rpm) of the mouse’s falling were registered automatically by the device software. For each mouse, the test was repeated three times with 1-min interval. The mean fall latency of the three trials was taken as the final parameter.

#### Morris water maze

MWM serves to test for spatial learning and memory and was carried out on a software–hardware complex designed in the Institute of Automation and Electrometry SB RAS (Novosibirsk) and adapted for SPF conditions (**Supplementary Figures S3A, B**) and following a 5-day testing pipeline, described earlier (42). During the acquisition phase for four consecutive days, mice were trained to find the platform. The following parameters were registered automatically: (1) latency time (s): the time for the mouse to find the platform location; (2) path (m) traveled by the animal from the moment it was placed into the water till it found the platform; and (3) cumulative distance (m) between the geometric center of the mouse and the platform. For each day, latency, path, and distance were calculated as the average of the three attempts.

On day 5, the retention test took place. The platform was removed and a mouse was placed at the center of the pool. Results were averaged over the three attempts. A statistically significant excess of the 25% of time spent in the target sector was considered as successful memorizing of the platform’s position.

#### Startle test

The test was conducted on the SR-Pilot Startle Response System (SR LAB, USA) following a previously described paradigm (48). The test consisted of six sessions, each of which included either a single pulse (P) or a pulse with a prepulse (PP). The magnitude of an acoustic startle response was measured with an accelerometer sensor starting 20 ms after the main signal. The average potential throughout the measurement was taken as the final parameter and prepulse inhibition was calculated as follows: PPI = (*A*_P_ − *A*_PP_)/*A*_P_ × 100%, where *A*_P_ is the amplitude of the reaction to the pulse and *A*_PP_ is the amplitude of the reaction to the pulse with the prepulse.

#### Home cage activity

Daily locomotor activity, sleep duration, and food and water consumption were assessed for 72 h with the PhenoMaster device (TSE, Germany) according to the manufacturer’s instruction and as described in detail elsewhere (42). The first 24 h (1–24 h) were considered as adaptive and were not taken into account. Home cage activity of hours 25–72 was analyzed and averaged for one representative 24 h. Locomotor activity is presented as the distance traveled during each hour (m). Sleep data are presented as cumulative sleep duration (min) during each hour. Food and water consumption is displayed as quantity in grams and milliliters, respectively, ingested during every 2 h.

#### Operant wall

The “operant wall” unit is a metal wall mounted in each individual cage of the PhenoMaster system (TSE, Germany) and is utilized to evaluate associative learning with the previously described paradigm (46). The operant wall was turned on from 15:30 to 17:30 during the mice’s presence in the PhenoMaster-equipped cage. As animals were not subjected to food deprivation, to arouse their interest and familiarize with the reward, a pellet was dispensed without any tasks at habituation day. During the next 3 days, to get the reward, the animal had to perform tasks. To assess the learning capabilities, the total number of received pellets and performed nose pokes were recorded during the task of days 2 and 3.

### 5-HT receptor functional activity

#### Quantification of 5-HT_1A_ receptor functional activity

The functional activity of the 5-HT_1A_ receptor was estimated by quantifying the hypothermic response obtained after acute administration of the 5-HT_1A_ agonist 8-OH-DPAT (1 mg/kg, i.p.) (49, 50). The body temperature was measured by means of a KJT thermocouple (Hanna Instruments, Singapore) with Cooper Constantan Rectal Probes for mice (Physitemp Instruments, Clifton NJ, USA) before the injection and 20 min after drug or saline administration.

#### Quantification of 5-HT_2A_ receptor functional activity

Head twitches in rodents are the main indicator of the activation of the 5-HT_2A_ receptor *in vivo* (51). A single administration of receptor 5-HT_2A_ agonist DOI (2,5-dimethoxy-4-iodoamphetamine) (1 mg/kg, i.p.) was performed. Five minutes after DOI treatment, head twitches were counted for 20 min.

#### Quantification of 5-HT_7_ receptor functional activity

The functional activity of the 5-HT_7_ receptor was evaluated as the intensity of the hypothermic response to the selective 5-HT_7_ agonist LP44 (4-[2-(methylthio)phenyl]-N-(1,2,3,4-tetrahydro-1-naphthalenyl)-1-piperazinehexanamide hydrochloride) (20.5 nM, i.c.v.) (49). Animals were anesthesized with isoflurane and administered LP44 diluted in sterile water into the left cerebral ventricle (i.c.v.) by microinjection using a stereotaxic instrument (TSE, Germany) at the following coordinates: AP –0.5, L –1.6 mm, DV 2 mm (52). Twenty minutes after injection, the body temperature offset was measured.

### Magnetic resonance imaging

The ^1^H MRI experiment was performed on a horizontal 11.7 T magnet (BioSpec 117/16 USR; Bruker, Germany) as described earlier (53). The brain structures were delineated using the ImageJ software (54) and The Allen Mouse Brain Atlas (55) by an experienced researcher blind to the group assignment (**Supplementary Figure S4**). Volumes of the brain structures and total volume of the brain were estimated using 23 slices of coronal orientation (slice thickness: 0.5 mm, inter-slice gap: 0 mm) and calculated as a sum of the areas of slices multiplied by 0.5 mm. The areas of structures in each slice were calculated as the number of pixels multiplied by the size of 1 pixel in square millimeters. The volumes of the measured structures were normalized to the total brain volume and are presented as a percentage (%) of the total brain volume.

### Biomolecular techniques

Brain structures including the frontal cortex, hippocampus, striatum, and midbrain were homogenized in 300 µL of Tris-HCl buffer (50 mM, pH 7.6) at 4°C using a mechanical homogenizer (Z359971, Sigma-Aldrich, USA). Aliquots of the homogenate were used for 5-HT and 5**-**HIAA content and TPH2 activity assays with chromatography as well as total RNA and total protein extraction.

#### 5-HT and 5-HIAA content assay

5-HT and 5-HIAA levels were measured in each brain structure with the previously described procedure (56) using a modular chromatographic analysis system (Shimadzu Corporation, USA) equipped with a Luna C18(2) column (5 μm particle size, L × I.D. 100 × 4.6 mm, Phenomenex, USA), a gradient pump (LC-20AD) with a vacuum degasser (DGU-20A5R), an autosampler with a 100-µL loop (SIL-20A), and an electrochemical detector (750 mV, DECADE II, Antec, Netherlands). The 5-HT and 5-HIAA contents were normalized to the amount of total protein measured by means of the Bradford method as described elsewhere (57) and are expressed in nanograms per 1 mg of total protein.

#### Tryptophan hydroxylase 2 activity assay

TPH2 activity was assessed using the modular chromatographic analysis system described above and following a previously reported method (57). The substrate of the reaction of L-tryptophan was present in the mixture in a concentration of 0.4 mM. Enzymatic activity was calculated as the amount of synthesized 5-hydroxytryptophan (pmol) per minute normalized to the amount of total protein in the sample.

#### Gene expression quantification with RT-PCR

Total RNA was extracted from the homogenate with the TRIzol Reagent (Life Technologies, USA) according to the manufacturer’s instructions and a previously described protocol (28). On the extracted mRNA, the reverse transcription reaction was performed to synthesize complementary DNA. Gene expression was measured via detection of the fluorescence of the intercalating dye SYBR Green I (R-402 Master Mix, Syntol, Russia). The utilized primers are presented in **Table 2**. Gene expression was measured using a two-standard method (58–60). As an external standard, a genomic DNA isolated from C57BL/6 male mouse hepatocytes was used (concentrations of 0.06, 0.125, 0.25, 0.5, 1, 2, 4, 8, 16, 32, and 64 ng/µL). A housekeeping gene, *Polr2a* (encoding a subunit of DNA-dependent RNA polymerase 2), served as an internal standard. Gene expression was evaluated as the number of complementary DNA copies of a target gene per 100 copies of *Polr2a*.

**Table 2:** The sequences of utilized primers, their annealing temperatures, and amplicon lengths.

Gene	Primer sequence	Annealing temperature, °C	Product length, bp
*rPol2a*	F 5′-tgtgacaactccatacaatgc-3′ R 5′-ctctcttagtgaatttgcgtact-3′	60	194
*Maoa*	F 5′-aatgaggatgttaaatgggtagatgttggt-3′ R 5′-cttgacatattcaactagacgctc-3′	64	138
*Tph2*	F 5′-cattcctcgcacaattccagtcg-3′ R 5′-cttgacatattcaactagacgctc-3′	61	239
*Htr1a*	F 5′-ctgtgacctgtttatcgccctg-3′ R 5′-gtagtctatagggtcggtgattgc-3′	62	109
*Htr2a*	F 5′-agaagccaccttgtgtgtga-3′ R 5′-ttgctcattgctgatggact-3′	61	169
*Htr7*	F 5′-ggctacacgatctactccaccg-3′ R5′-cgcacactcttccacctccttc-3′	65	198
*Slc6а4*	F 5′-cgctctactacctcatctcctcc-3′ R 5′-gtcctgggcgaagtagttgg-3′	63	101

#### Protein quantification with Western blot analysis

For the assessment of protein levels, the homogenate was prepared for Western blot analysis as described earlier (28). The extracts (20 µg per lane) were resolved on a 10% sodium dodecyl sulfate (SDS) polyacrylamide gel and blotted onto a nitrocellulose membrane. The antibody used for target protein detection and the detected protein weights are listed in **Table 3**. Target protein quantities were normalized to the GAPDH protein level and expressed in relative units.

**Table 3:** Antibody utilized in the Western blot analysis, their producers, used dilutions, and targets’ molecular weights.

Antigen	Dilution	Ref. no. and manufacturer	Molecular weight, kDa
STEP	1:1,000 in 5% BSA	sc-23892, Santa Cruz Biotechnology, USA	61, 46, 38, 33
pERK1/2	1:2,000 in 5% milk	Phospho-p44/42 MAPK (Thr202/Tyr204) Antibody #9101, Cell Signaling, USA	42, 44
TPH2	1:1,000 in 5% milk	ab184505, Abcam, UK	56
MAOA	1:500 in 5% milk	ab126751, Abcam, UK	60
5-HT_1A_	1:500 in 5% milk	ab85615, Abcam, UK	60
5-HT_2A_	1:1,000 in 5% FBS	sc-15073, Santa Cruz Biotechnology, USA	52
5-HT_7_	1:1,000 in TBST	ab128892, Abcam, UK	52
5-HTT	1:500 in 5% FBS	303614, US Biological Life Sciences, USA	41
GAPDH	1:2,000 in 5% FBS	ab9485, Abcam, UK	37

### Statistical analyses

Home cage behavior monitored parameters: locomotor activity, sleep duration, and food and water consumption were analyzed with repeated-measures ANOVA with factors “Genotype” and “Hour” and are presented as means ± standard deviation (SD) for each hour (locomotor activity and sleep duration) or 2 h (food and water consumption). The Fisher *post-hoc* analysis was utilized to determine the significance of differences between genotypes for each hour/2 h.

Repeated-measures ANOVA with the Fisher *post-hoc* analysis was likewise used to analyze the behavior of mice in the learning phase of the MWM test with factors “Genotype” and “Day”. Repeated-measures ANOVA was then performed for each genotype separately with the factor “Day”, followed by the Bonferroni correction, to assess the learning performance of each mouse strain. Test day performance was analyzed with the Student’s *t*-test *vs*. the 25% value, subject to the Bonferroni correction.

The rest of the behavioral parameters as well as brain structure volumes, monoamine content, gene expression, protein levels, TPH2 activity, and receptors’ functional activity were tested for normality and equality of dispersion with Lilliefors’ and Barlett’s tests, respectively. Normal and non-normal distributions were then analyzed with the Student’s *t*-test or Mann–Whitney *U*-test, respectively. Data are presented as means ± SD.

## Results

### Genotyping of the knockout mouse strain

The *Ptpn5* gene has four isoforms produced by alternative splicing and their corresponding proteins: STEP61, STEP46, STEP38, and STEP20. Two of these forms, STEP61 and STEP46, contain a PTP sequence required for catalytic activity. C57BL/6-Ptpn5_KO knockout mice were generated using the CRISPR/Cas9 system. As a result, 28 pups were delivered by foster females and genotyped with PCR. Six of them had a deletion of the expected size. Pup #11 was selected as the founder for the generation of the *Ptpn5* gene knockout mice; the 347-bp deletion (chr 7:46728942–46729288, GRCm39/mm39) was confirmed by Sanger sequencing. The deleted genomic region consists of 94 bp of the *Ptpn5* exon 12 and the complete exon 13 (114 bp) and includes the PTP catalytic domain (**Figure 1B**). The analysis of the STEP protein level in the striatum using Western blot analysis showed the absence of signal in *Ptpn5* mutant mice, indicating a potential STEP protein structure disruption and absence of this protein in knockout mice (**Figure 1C**).

### Mutant STEP protein demonstrates lack of function

STEP46 and STEP61 isoforms dephosphorylate ERK1/2 kinases, thereby modulating several protein signaling pathways in the cell. Western blot analysis revealed an elevated ERK1/2 phosphorylation level in the striatum of *Ptpn5* KO mice compared to the wild-type strain, which confirmed the reduction of STEP phosphatase activity in knockout mice (**Figures 1D, E**). Deletion in the *Ptpn5* gene resulted in a significantly higher level of pERK2 protein in the striatum (*t*_14_ = 2.25, *p* < 0.05), while the elevation of pERK1 level was insignificant (*t*_13_ = 1.94, *p* = 0.07).

### Behavioral phenotyping

#### Home cage behavior

**Figure 2:**
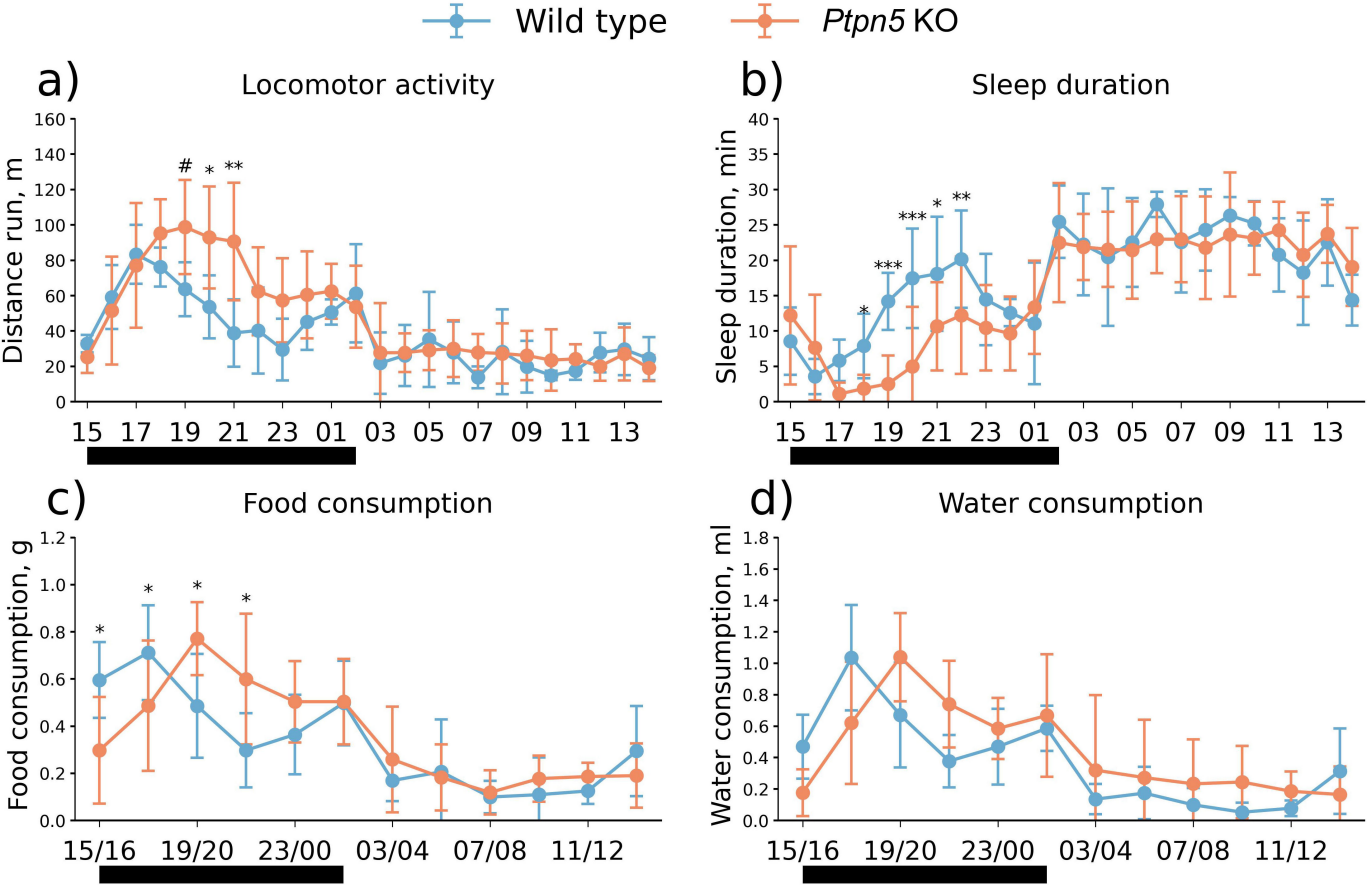
The daily dynamics of **(a)** locomotor activity (distance run, m) and **(b)** cumulative sleep duration (min) were continuously measured over each hour; **(c)** food and **(d)** water consumption were measured every 2 h in the home cage of the *Ptpn5* KO and wild-type mice. The data were averaged over 2 days of monitoring. The *X*-axis shows day time (h). Black bars under the *X*-axis highlight the dark phase of the day. Indicated statistical significance refers to the difference between genotypes for the corresponding hour. *p < 0.05, **p < 0.01, ***p < 0.001, ^#^*p* = 0.059 compared to the wild type (eight animals per group). Groups were compared with repeated-measures ANOVA and Fisher *post-hoc* analysis for each hour/2 h.

A significant effect of the *Ptpn5* knockout on the home cage behavior was found. *Ptpn5* KO mice were more active as indicated by the elevated locomotor activity (genotype effect: *F*_1,14_ = 6.64, *p* < 0.05; genotype × hour interaction: *F*_23,322_ = 3.41, *p* < 0.001) (**Figure 2A**) and reduced sleep duration (genotype effect: *F*_1,14_ = 16.11, *p* < 0.01; genotype × hour interaction: *F*_23,322_ = 2.45, *p* < 0.001) (**Figure 2B**). The main differences occurred during the dark phase (active period of mice), between hours 19 and 21 for the distance traveled and between hours 18 and 22 for the cumulative sleep duration. No genotype effect was detected on the average food (*F*_1,14_ < 1) (**Figure 2C**) or water consumption (*F*_1,14_ = 1.13, *p* > 0.05) (**Figure 2D**). At the same time, the interaction of factors genotype and hour was significant for these parameters (food consumption: *F*_11,154_ = 4.38, *p* < 0.001; water consumption: *F*_11,154_ = 4.18, *p* < 0.001). Nonetheless, no differences for distinct hours were present for water consumption. Food consumption was higher for wild-type mice during hours 15–18 and greater for the *Ptpn5* KO mice for the four subsequent hours.

#### Operant wall

Associative learning tested in the “operant wall” paradigm was not affected by the *Ptpn5* gene knockout. No effect was detectable for both the number of obtained pellets and the number of nose pokes (**Supplementary Table S1**).

#### Open field test

In the OF test, no effects of the *Ptpn5* gene knockout were observed on the total distance traveled (locomotor activity), time spent in the center of the arena (**Supplementary Figures S1C, D**), and the duration of rearing (exploratory activity). Meanwhile, in the knockout mice, the duration of grooming behavior was increased compared to wild-type mice (*U* = 9, *p* < 0.05), indicating elevated displacement activity (**Supplementary Table S1**).

#### Forced swim test

**Figure 3:**
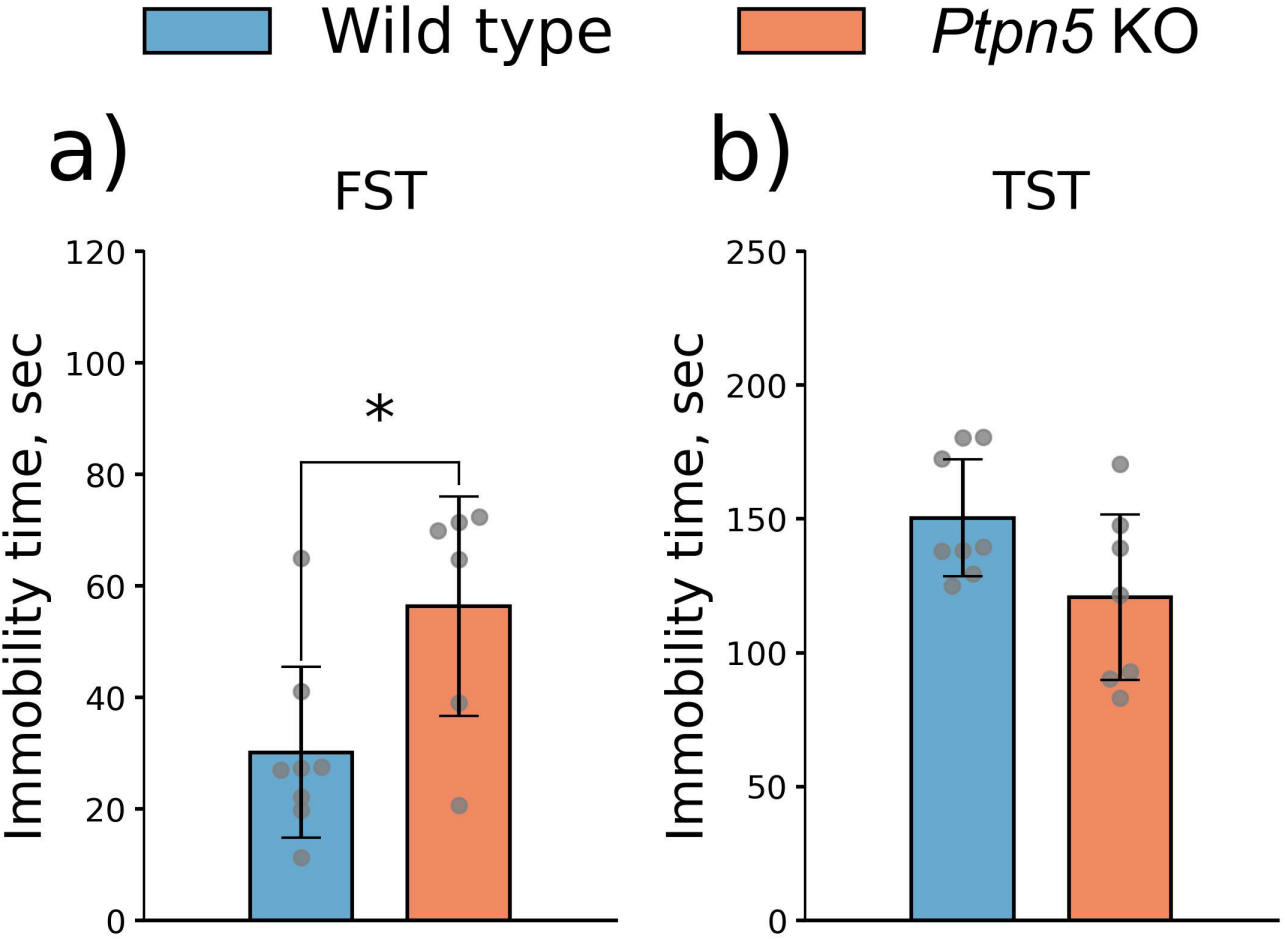
Effects of the *Ptpn5* gene knockout on behavior of mice in the **(a)** forced swim test and **(b)** tail suspension test. *p < 0.05 compared to the wild type (six to eight animals per group). Groups were compared with *t*-test for independent samples.

In the FST, *Ptpn5* KO mice demonstrated elevated total immobility time (*t*_12_ = 2.59, *p* < 0.05) (**Figure 3A**).

#### Tail suspension test

In the TST, we observed a trend toward a reduction of immobility in the *Ptpn5* KO mice compared to wild-type animals (*t*_13_ = 2.02, *p* > 0.05) (**Figure 3B**).

#### Marble burying test

**Figure 4:**
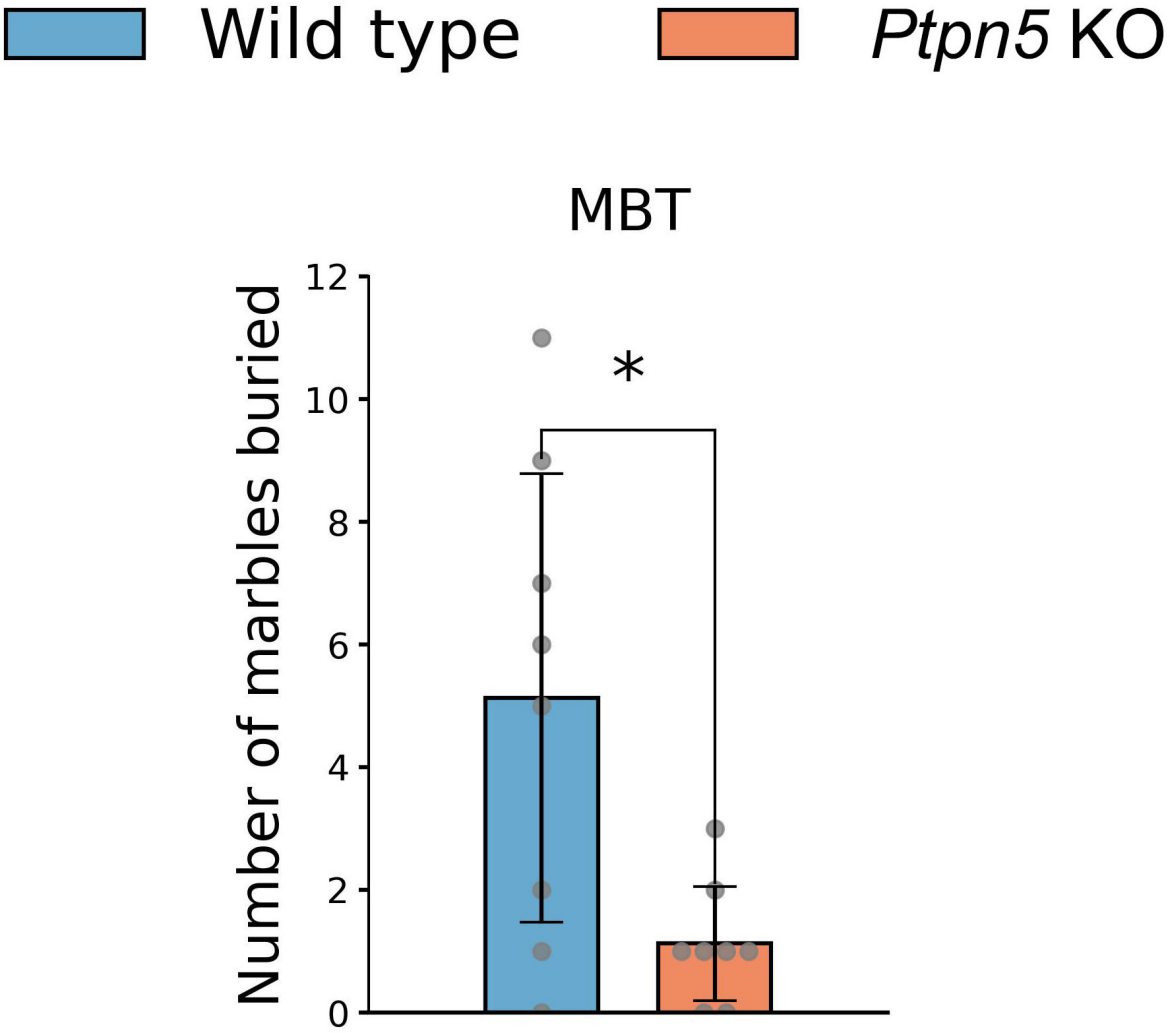
Effects of the *Ptpn5* gene knockout on behavior of mice in the marble burying test. *p < 0.05 compared to the wild type (eight animals per group). Groups were compared with the Mann–Whitney *U*-test.

In the MBT, *Ptpn5* KO mice buried significantly fewer marbles than the wild-type genotype, indicating attenuated stereotypic and anxiety-related behavior (*U* = 12.5, *p* < 0.05) (**Figure 4**).

#### Novel object test

In the NOR test, the total time of contact with the novel object was similar in both strains of mice (**Supplementary Table S1**).

#### Social behavior in the social interaction and three-chambered tests

The social behavior of *Ptpn5* KO mice did not differ from wild-type mice in the social interaction (**Supplementary Table S1**) and three-chambered tests (**Supplementary Table S1**).

#### Elevated plus-maze test

**Figure 5:**
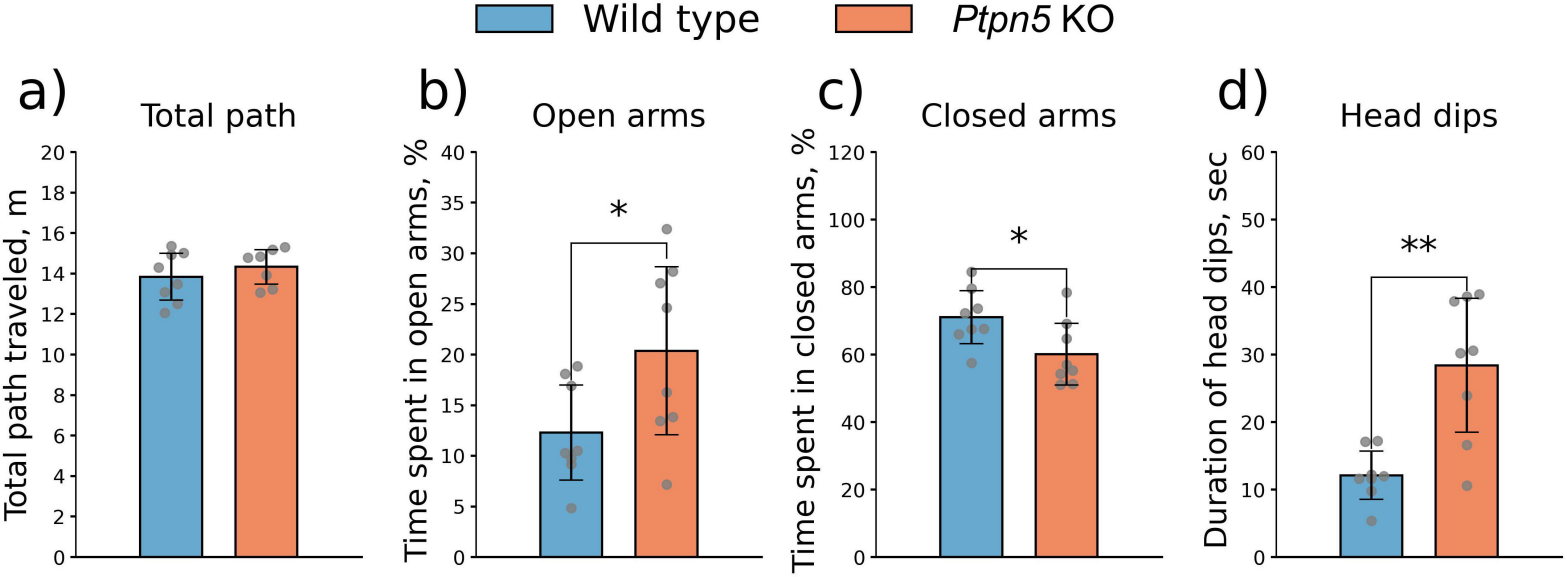
Effects of the Ptpn5 gene knockout in mice on the **(a)** total path traveled, time spent in the **(b)** open and **(c)** closed arms, and **(d)** duration of head dips in the elevated plus maze test. *p < 0.05, **p < 0.01 compared to the wild type (seven to eight animals per group). Groups were compared with *t*-test for independent samples.

In the EPM, *Ptpn5* KO mice exhibited diminished anxiety-like behavior, spending more time in the open arms (*t*_14_ = 2.24, *p* < 0.05) (**Figure 5B**) and less time in the closed arms (*t*_14_ = 2.41, *p* < 0.05) (**Figure 5C**) compared to the wild type (**Supplementary Figures S2C, D**). Moreover, mice with the mutation showed elevated exploratory activity and risk assessment, as indicated by a higher number of head dips (*t*_14_ = 4.09, *p* < 0.01) (**Figure 5D**). Meanwhile, no difference between strains was documented in the overall activity (total path traveled) (*t*_13_ = 0.86, *p* > 0.05) (**Figure 5A**), duration of stretch postures, or time spent in the center of the maze (**Supplementary Table S1**).

#### Rotarod test

No changes in the motor function were observed in the rotarod test, as the latency to fall from the rod was equivalent in both strains of mice (**Supplementary Table S1**).

#### Startle test

**Figure 6:**
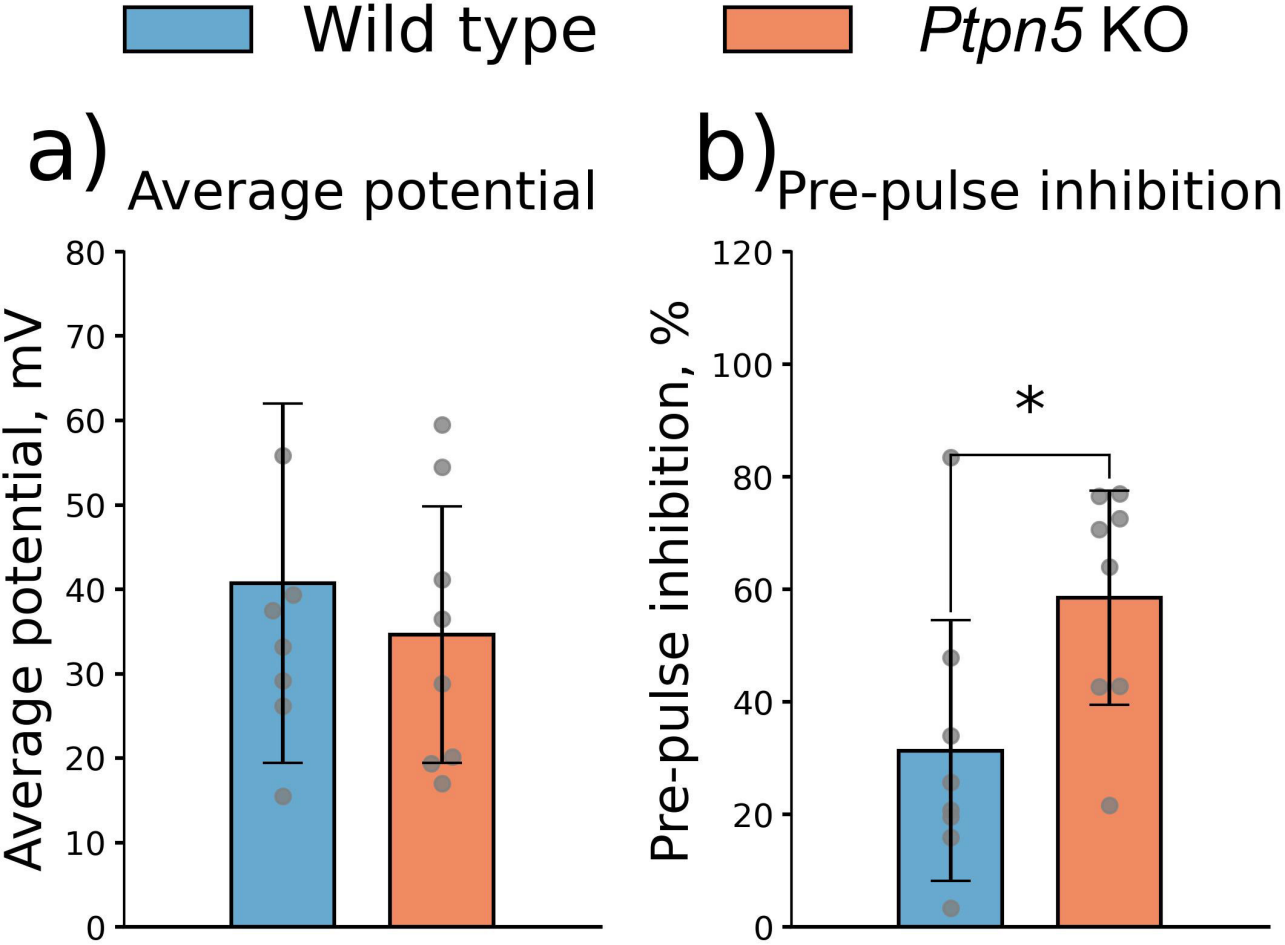
Effects of the *Ptpn5* gene knockout on behavior of mice in the startle test. **(a)** Average potential of pulse and prepulse, **(b)** prepulse inhibition, calculated as PPI = (P−PP)/P * 100%, where P: reaction to pulse registered as average potential, PP: reaction to pulse with prepulse registered as average potential. *p < 0.05 compared to the wild type (eight animals per group). Groups were compared with *t*-test for independent samples.

In the startle response test, the *Ptpn5* KO mice displayed a more profound prepulse inhibition evaluated by the average measured potential (*t*_14_ = 2.40, *p* < 0.05) (**Figure 6B**). The average potential amplitude did not differ between genotypes (*t*_14_ = 0.62, *p* > 0.05) (**Figure 6A**).

#### Morris water maze

**Figure 7:**
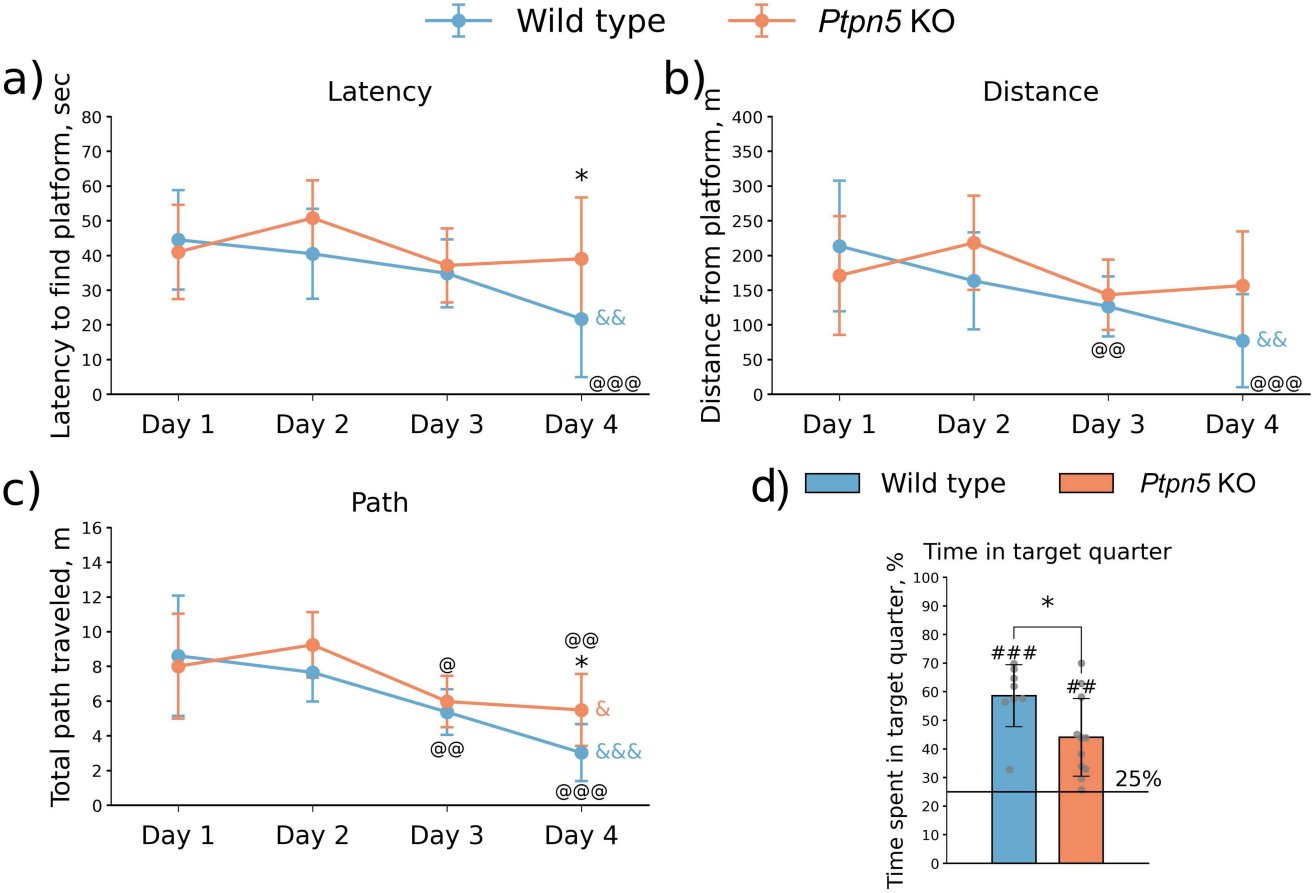
Effects of the *Ptpn5* gene knockout on behavior of mice in the **(a–c)** learning phase and **(d)** retest session of the Morris water maze. ^@^*p* < 0.05, *p* < 0.01, ^@^*p* < 0.001 compared to Day 1; ^&^*p* < 0.05, ^&&^*p* < 0.01,^&&&^*p* < 0.001 effect of Day; *p < 0.05 compared to the wild type; ^##^*p* < 0.01, ^###^*p* < 0.001 compared to 25% (seven to eight animals per group). **(a–c)** Groups were compared with repeated-measures ANOVA with Fisher *post-hoc* analysis for each day, **(d)** groups were compared against 25% with *t*-test for single means and with each other with *t*-test for independent samples.

In the learning phase of the MWM test, we found no effect of the factor Genotype (latency to find the platform: *F*_1,17_ = 2.65, *p* > 0.05; distance traveled: *F*_1,17_ = 3.47, *p* > 0.05; cumulative distance: *F*_1,17_ = 1.77, *p* > 0.05) or the genotype × day interaction on the distance traveled (*F*_3,51_ = 1.76, *p* > 0.05) (**Figure 7C**). At the same time, there was a significant effect of the factor Day on all of the measured parameters (latency to find the platform: *F*_3,51_ = 6.03, *p* < 0.01; distance traveled: *F*_3,51_ = 17.14, *p* < 0.001; cumulated distance: *F*_3,51_ = 6.53, *p* < 0.001). Moreover, a significant effect of the genotype × day interaction was observed in the cumulative distance (*F*_3,51_ = 3.08, *p* < 0.05), and a trend was noted in the latency to find the platform (*F*_3,51_ = 2.66, *p* = 0.058). Wild-type mice swam closer to the platform with each learning day (*F*_3,21_ = 8.41, *p* < 0.01), whereas no such improvement was detected for the *Ptpn5* KO group (*F*_3,27_ = 1.61, *p* > 0.05) (**Figure 7B**). A similar tendency was observed for the latency to find the platform (wild type: *F*_3,21_ = 6.92, *p* < 0.01; *Ptpn5* KO: *F*_3,27_ = 1.86, *p* > 0.05) (**Figure 7A**, **Supplementary Figure S3C, D**).

On the fifth day during the retest session, the spatial memory was assessed. Both strains spent more than 25% of the time in the target area (wild type: *t*_7_ = 8.23, *p* < 0.001; *Ptpn5* KO: *t*_10_ = 4.44, *p* < 0.01), which indicates that animals remembered the position of the platform. However, wild-type mice spent more time in the target quadrant of the maze compared to the *Ptpn5* KO animals (*t*_17_ = 2.37, *p* < 0.05) (**Figure 7D**, **Supplementary Figure S3E, F**).

### MRI

#### Brain structure volume

Structural MRI analysis revealed significant differences in the volume of distinct brain regions between wild-type mice and *Ptpn5* KO mice. Mutant mice were characterized by a greater volume of the cortex and striatum, whereas the midbrain and cerebellum were smaller in the *Ptpn5* KO mice compared to the wild-type animals (**Table 4**). At the same time, no differences were noticed in the volume of the whole brain, hippocampus, interbrain, and pituitary between the strains (**Table 4**).

**Table 4:** Effects of the *Ptpn5* gene knockout on total volumes of brain structures in mice (8–11 animals per group).

Brain structure	Wild type	*Ptpn5* KO	Statistical values
Whole brain	476.37 ± 32.52	491.2 ± 29.66	*t*_17_ = 1.03, *p* > 0.05
Cortex	26.94 ± 0.52	27.96 ± 0.76	*t*_17_ = 3.28, **p < 0.01
Striatum	8.78 ± 0.32	9.64 ± 0.61	*t*_17_ = 3.6, **p < 0.01
Hippocampus	6.61 ± 0.43	6.46 ± 0.32	*t*_17_ = 0.89, *p* > 0.05
Interbrain	8.33 ± 0.84	8.57 ± 1.04	*t*_17_ = 0.53, *p* > 0.05
Midbrain	9.87 ± 1.07	9.02 ± 0.66	*t*_17_ = 2.11, *p < 0.05
Cerebellum	13.99 ± 0.91	12.84 ± 1.11	*t*_17_ = 2.4, *p < 0.05
Pituitary	0.38 ± 0.08	0.38 ± 0.04	*t*_17_ = 0.06, *p* > 0.05

Whole brain volume is presented in mm^3^, volumes of other brain regions are presented as % of the whole brain volume. Groups were compared with *t*-test for independent samples.

Statistically significant differences are highlighted in bold.

### Serotonin system

#### 5-HT and 5-HIAA levels

**Figure 8:**
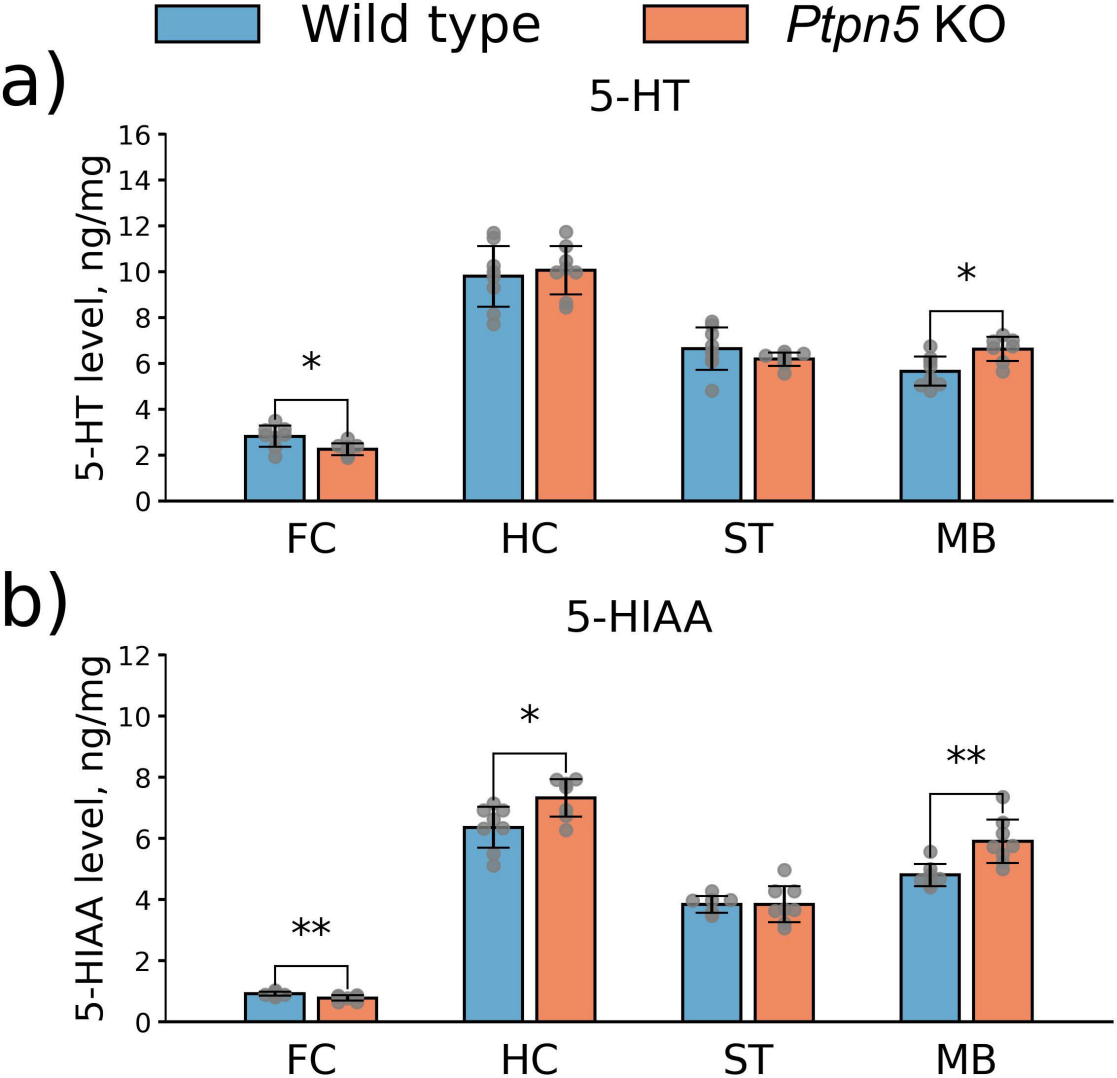
Effects of the *Ptpn5* gene knockout on the **(a)** 5-HT and **(b)** 5-HIAA levels in the frontal cortex (FC), hippocampus (HC), striatum (ST), and midbrain (MB) of mice, presented as amount of 5-HT or 5-HIAA (ng) per 1 mg of total protein in the probe. *p < 0.05, **p < 0.01 compared to the wild type (seven to eight animals per group). Groups were compared with *t*-test for independent samples.

*Ptpn5* KO mice displayed diminished 5-HT (*t*_14_ = 2.87, *p* < 0.05) (**Figure 8A**) and (its metabolite) 5-HIAA (*t*_12_ = 3.15, *p* < 0.01) (**Figure 8B**) levels in the frontal cortex compared to the wild-type mice and elevated levels of these substances in the midbrain (5-HT: *t*_13_ = 2.96, *p* < 0.05; 5-HIAA: *t*_13_ = 3.45, *p* < 0.01) (**Figures 8A, B**). 5-HT and 5-HIAA content in the striatum was not affected by the *Ptpn5* gene knockout (5-HT: *t*_14_ = 1.27, *p* > 0.05; 5-HIAA: *t*_13_ = 0.05, *p* > 0.05) (**Figures 8A, B**), whereas in the hippocampus, *Ptpn5* KO mice showed an increase in the level of 5-HIAA (*t*_13_ = 2.69, *p* < 0.05) (**Figure 8B**) with unchanged 5-HT content (*t*_14_ = 0.41, *p* > 0.05) (**Figure 8A**). Meanwhile, no difference between genotypes was documented in the serotonin metabolism index (5-HIAA/5-HT) in any of the investigated brain structures (**Supplementary Table S2**).

#### Key enzymes of serotonergic system

**Figure 9:**
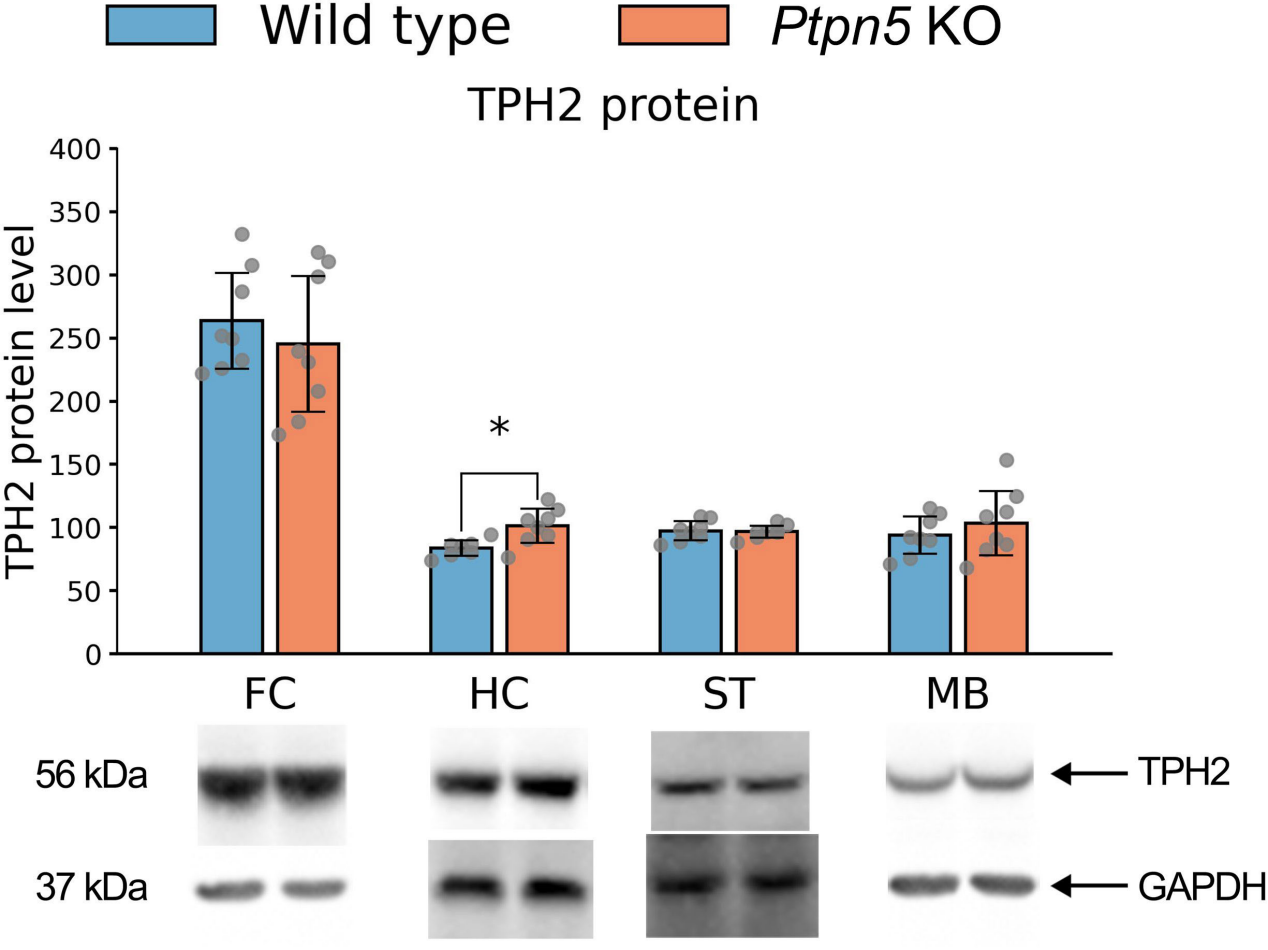
Effects of the *Ptpn5* gene knockout on the TPH2 protein level in the frontal cortex (FC), hippocampus (HC), striatum (ST), and midbrain (MB) of mice, presented as the percentage of the GAPDH protein level. *p < 0.05 compared to the wild type (seven to eight animals per group). Groups were compared with *t*-test for independent samples.

Enzymatic activity of TPH2 did not differ between genotypes (**Supplementary Table S3**). *Tph2* gene expression was also not affected by the *Ptpn5* gene knockout (**Supplementary Table S3**). However, we have detected significant differences in the TPH2 protein level in the hippocampus (*t*_13_ = 2.97, *p* < 0.05): *Ptpn5* KO mice showed an upregulated level of this protein in this brain region, but not in the frontal cortex, striatum, or midbrain (**Figure 9**). No significant differences were registered in the *Maoa* gene expression or MAOA protein levels in either of the investigated brain structures (**Supplementary Table S4**).

#### Expression of serotonin transporter

*Ptpn5* gene knockout did not affect the serotonin transporter expression in the frontal cortex, hippocampus, midbrain, or striatum (**Supplementary Table S5**).

#### Expression and functional activity of 5-HT_1A_, 5-HT_2A_, and 5-HT_7_ receptors

**Figure 10:**
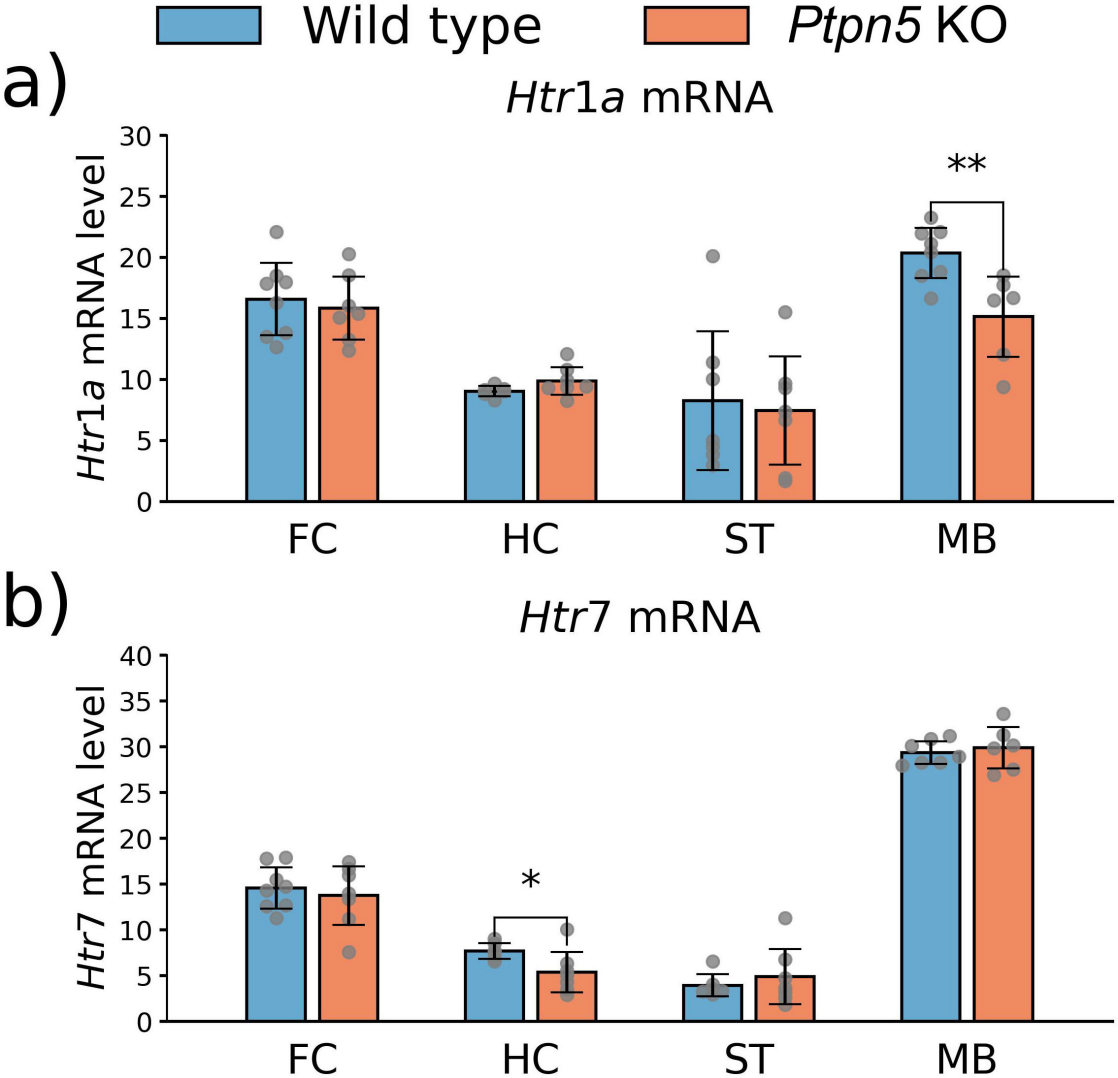
Effects of the *Ptpn5* gene knockout on the **(a)***Htr1a* and **(b)***Htr7* genes’ expression in the frontal cortex (FC), hippocampus (HC), striatum (ST), and midbrain (MB) of mice, evaluated as the number of transcript copies per 100 copies of *Polr2a* mRNA. *p < 0.05, **p < 0.01 compared to the wild type (six to eight animals per group). Groups were compared with *t*-test for independent samples.

Mice with a *Ptpn5* gene mutation were characterized by a reduction in *Htr1a* mRNA level in the midbrain (*t*_12_ = 3.36, *p* < 0.01) (**Figure 10A**) and by a reduction in *Htr7* mRNA level in the hippocampus (*t*_11_ = 2.22, *p* < 0.05) (**Figure 10B**) compared to the wild type. No differences in *Htr2a* gene expression were unveiled in all studied structures (**Supplementary Table S7**). Moreover, no changes in *Htr1a* gene expression were found in the hippocampus, frontal cortex, and striatum (**Figure 10A**), as well as in *Htr7* mRNA levels in the frontal cortex, midbrain, and striatum (**Figure 10B**). 5-HT_1A_, 5-HT_2A_, and 5-HT_7_ protein levels and functional activities did not differ between the genotypes (**Supplementary Tables S6, S7, and S8**).

## Discussion

Phosphatase STEP is an important signal transduction protein in the neuron; its functions encompass a wide range of crucial processes, such as the regulation of synaptic function, long-term potentiation and depression, and cell death. Therefore, STEP participates in behavioral control, and its dysregulation is associated with numerous neurodegenerative disorders. It is well-known that the 5-HT system takes part in the regulation of many types of behavioral traits and cognitive processes. However, the association of the serotonergic system and the phosphatase STEP is still poorly studied. In this paper, we aimed to fill this gap using *Ptpn5* gene knockout mice generated using the CRISPR/Cas9 system and excising the PTP-domain-encoding sequence. Previously, *Ptpn5* KO mice have been created by Venkitaramani et al. (61) utilizing homologous recombination and replacing the PTP-domain sequence with the neomycin cassette, thus adding exogenous genetic material and disrupting the open reading frame. This strain is characterized by behavioral alterations (25, 27), potentially associated with the 5-HT-system. Nevertheless, the 5-HT system changes as well as brain morphology in these mice have not been studied.

The designed mutation was intended to cleave the PTP-domain and produce an inactive STEP form but ultimately resulted in the complete absence of STEP protein. Perhaps, the misfolded structure is detected and destroyed by the cell’s proteasome system. Nonetheless, we succeeded in diminishing STEP activity in the brain, as indicated by the significant upregulation of phosphorylation in STEP substrates (ERK1/2 kinases). The lack of STEP protein caused significant changes in the brain morphology, behavior, and the 5-HT system. At the same time, *Ptpn5* KO mice are viable, healthy, fertile, and visually indistinguishable from their wild-type counterparts.

STEP dysfunction significantly affected the volume of several brain regions measured using structural MRI. The cortex and striatum, structures normally rich in *Ptpn5* mRNA (1), exhibited excessive growth, whereas midbrain and cerebellum, where *Ptpn5* expression is low or undetectable (1), were smaller compared to those of wild-type mice. On the one hand, STEP regulates the membrane localization of glutamate receptors, protecting neural cells from glutamatergic toxicity and overexcitation (62, 63). The absence of STEP in our mice may have disrupted this balance, potentially leading to the hypergrowth of certain structures. On the other hand, STEP also blocks the p38 kinase (64), which regulates apoptosis signaling cascades, and the absence of STEP would lead to the overactivation of this pathway and the elevation of cell death probability and shrinkage of nervous tissue. These results point to the importance of a balance in the STEP-dependent pathways for the development of brain morphology and architecture.

*Ptpn5* KO mice, in resemblance to the *Ptpn5* KO line previously obtained elsewhere (25, 33), did not demonstrate any alterations in general locomotor activity, motor function, and exploratory or social behavior in a series of behavioral tests. In the home cage, however, *Ptpn5* KO mice were more active, had less sleep, and consumed more food between 18:00 and 22:00. The time span of these differences coincides with the end of the operant wall tasks, which took place from 15:30 to 17:30. While no significant differences were found in the task performance, wild-type mice received slightly more pellets on the second day than the *Ptpn5* KO mice and, perhaps, were less hungry and less active during the following hours.

*Ptpn5* KO mice showed decreased anxiety-like behavior in the EPM test, spending more time in the open arms and less time in the closed arms of the apparatus. While Blázquez and colleagues did not observe any differences in these parameters, their *Ptpn5* KO mice exhibited smaller latency to enter the open arms, indicating a slight anxiolytic action of the knockout (27). The anxiolytic effect of STEP inactivation agrees with the studies of the STEP inhibitor benzopentathiepin TC-2153 on mice, rats, and fish (28, 65–67). Furthermore, highly aggressive rats, which have elevated levels of STEP protein in the brain, are characterized by higher anxiety compared to their tame counterparts (28). In the MBT, *Ptpn5* KO mice buried significantly less marbles, which, together with decreased stereotypy, can be indicative of lower proneness to anxiety. The MBT is widely used to model autistic-like behavior in rodents (44), and the inhibition of STEP with TC-2153 also attenuates behavioral traits associated with this type of behavior (68). However, in the present study, we did not find any disturbances in social behavior characteristic of the autistic phenotype, so we suppose that the observations in the MBT are rather associated with the general anxiolytic effect of the knockout.

In the startle response test, *Ptpn5* KO mice exhibited an elevated pre-pulse inhibition index compared to the wild-type controls. Pre-pulse inhibition deficit is usually associated with schizophrenia (69). Upregulated STEP protein levels have been documented in the pharmacological models of this pathology both in the brains of mice and in the cell cultures (31, 34). A STEP inhibitor alleviated behavioral and molecular abnormalities in these models (31, 34). In the current study, the PPI improvement can possibly be associated with the overdeveloped cortex and striatum (69, 70) and therefore enhanced sensorimotor gating control (71). Of note, hyperactivation of the orbitofrontal cortex–ventromedial striatum system has been reported to increase grooming behavior in mice (72). In the OF test, we revealed elevated displacement activity in *Ptpn5* KO mice indicated by more pronounced grooming behavior, which also coincides with previously obtained results (27).

Mutant mice performed poorly in the water-related tests: FST and MWM. In the MWM, *Ptpn5* KO mice experienced difficulty finding the platform. In contrast to the control group, the cumulative distance from the platform and the time latency to find the platform did not improve for the *Ptpn5* KO mice throughout the learning phase. Meanwhile, the total traveled path did decrease on the 4th day compared to day 1. The observed pattern can indicate an increase of immobility after a long period of being subjected to stressful conditions. In support of this idea, the immobility time in the FST was also prolonged in mutant mice, whereas the analogous but dry TST did not reveal any differences between strains. Therefore, we hypothesize that the observed changes are rather associated with reaction to environmental stress than depressive-like behavior or deficiency in learning ability. On the retest day in the MWM, *Ptpn5* KO mice remembered the location of the platform, but still spent less time in the target quarter than the control group, which may also be linked to the immobility reaction to water. At the same time, in the TST (related to depressive-like behavior), the novel object recognition test (memory), and the operant wall task (learning ability), we did not detect any differences between strains.

The above-discussed behavioral traits are, in particular, regulated by the brain 5-HT system (12). Here, we show that *Ptpn5* knockout induced changes in different components of this system. First of all, we detected significant alterations in the content of 5-HT and its metabolite in various brain regions. In the midbrain of *Ptpn5* KO mice, these substances were upregulated, whereas in the frontal cortex, both were diminished compared to the wild-type mice. In the hippocampus, we observed only a rise in the 5-HIAA level and no changes were found in the striatum. Furthermore, the lack of STEP led to the accumulation of TPH2 protein in the hippocampus of *Ptpn5* KO mice. We did not register any differences in the mRNA levels, which could mean that, to the greatest extent, the translation or degradation processes of this protein were affected. The mutation did not alter MAOA or 5-HTT expression, but attenuated the expression of *Htr1a* gene in the midbrain and *Htr7* gene in the hippocampus.

Several mechanisms of *Ptpn5* KO that affect the 5-HT system could have taken place. Structural MRI revealed a decrease in total midbrain volume, which is a central brain region for the 5-HT system, and home to the 5-HT nuclei (73). Moreover, we detected lower expression of *Htr1a* mRNA in this structure. 1A receptors play a crucial role in the 5-HT system autoregulation (74–76); thus, this result can be associated with the observed elevation of serotonin and its metabolite levels in this structure due to the downregulation of presynaptic 1A receptors. STEP inhibitor TC-2153 had the same effect on the *Htr1a* gene expression after chronic administration to ASC mice (19) and acutely increased both 5-HT and 5-HIAA levels in the hypothalamus of C57BL/6 mice (17).

On the molecular level, the lack of STEP could have induced changes in the 5-HT system through glutamate signaling, the MAPK cascade, or neurotrophic factors. STEP directly dephosphorylates subunits of glutamate receptors (7, 8) and the interplay between these and 5-HT has been documented previously (77, 78). 5-HT receptors of the G protein-coupled receptors family can exert a downstream action on the MAPK pathway (79). Alterations in the functioning of one of the components of this signaling cascade—STEP substrates kinases ERK1/2—could lead to the dysregulation of the feedback signal and receptor desensitization and internalization mechanisms. An observed elevation of TPH2 protein level in the hippocampus of *Ptpn5* KO mice with no difference in the *Tph2* gene expression and TPH2 enzymatic activity could be explained by the improved protein stability of this enzyme. Although TPH2 does not contain any known tyrosine phosphorylation sites and therefore cannot be directly affected by STEP, its stability is known to be regulated by the serine-19 phosphorylation site (80). One of the key effectors of this site is Ca^2+^/calmodulin-dependent protein kinase type II (80), whose elevated activity was observed in the synaptosomes purified from *Ptpn5* KO mice (30).

The 5-HT system is one of the substantial regulators of anxiety-like behavior. The prefrontal cortex is involved in the top-down control of emotions and behavioral reactions, particularly anxiety (81, 82). *Ptpn5* KO mice were characterized by a greater volume of the cortex and a decreased level of 5-HT in the frontal cortex, which is in agreement with decreased anxiety, as this type of behavior has been observed in mice deficient in brain serotonin (83). We have noticed a reduction in *Htr7* mRNA levels in the hippocampus of *Ptpn5* KO mice, which could also have contributed to the anxiety attenuation since 5-HT_7_ receptor antagonists are known to produce an anxiolytic effect (84, 85). This interpretation is consistent with our results in aggressive and tame rats: the more anxiety-prone aggressive rats had higher *Htr7* mRNA levels in the frontal cortex and hippocampus (20). Meanwhile, we did not detect concomitant changes in the functional activity or protein levels of this receptor, so its role in the observed behavioral alterations is likely not primary. In turn, elevated levels of TPH2 protein in the hippocampus can be associated with the reduction of marble burying behavior of *Ptpn5* KO mice, as *Tph2* gene knockout mice tend to bury more marbles than the wild type (86).

Several observed 5-HT system peculiarities could have supported the PPI index strengthening in *Ptpn5* KO mice. It has been documented that the monoaminergic neurotoxin 5,7-dihydroxytryptamine, which affects 5-HT neurons, leads to PPI disruption (87). The TPH2 inhibitor para-chlorophenylalanine has a similar effect (87). Both these actions correspond to a 5-HT system deficit. The *Ptpn5* KO mice, on the other hand, exhibit higher levels of 5-HT in the midbrain and TPH2 protein in the hippocampus and therefore show PPI increase. Furthermore, 5-HT receptors could have taken part in these processes as well. The 5-HT_1A_ receptor agonist 8-OH-DPAT aggravates the PPI index (88, 89), and 5-HT_7_ antagonists exert a positive action on this parameter in pharmacological schizophrenia models (90, 91). *Ptpn5* KO mice showed reduced mRNA expression of these receptors in the midbrain and hippocampus, respectively, which therefore could have had a positive effect on PPI. Once again, we did not observe any differences in the protein levels or receptors’ functional activity; thus, their role is rather suggestive and requires further investigation.

## Conclusion

The excision of the PTP-motif-encoding sequence from the mouse *Ptpn5* gene resulted in a lack of STEP protein in the brain. It produced noticeable alterations in brain morphology, behavior, and the 5-HT system. These results once again point to the importance of the kinase-phosphatase balance for brain development, behavioral control, and the interplay between mediator systems. Essentially, this study provides a crucial verification of the STEP and 5-HT system interconnection, which offers a new perspective on the role of STEP in the pathogenesis of psychiatric disorders.

## Data Availability

The raw data supporting the conclusions of this article will be made available by the authors, without undue reservation.
