# Supplementary material for: Lack of striatal-enriched protein tyrosine phosphatase affected the serotonin system, behavior, and brain morphology in mice
Source: Front Psychiatry. 2026 Jan 14;16:1730197. doi: 10.3389/fpsyt.2025.1730197 (PMC12848919; doi:10.3389/fpsyt.2025.1730197)
Supplement: Supplementary file 1 [file DataSheet1.docx]

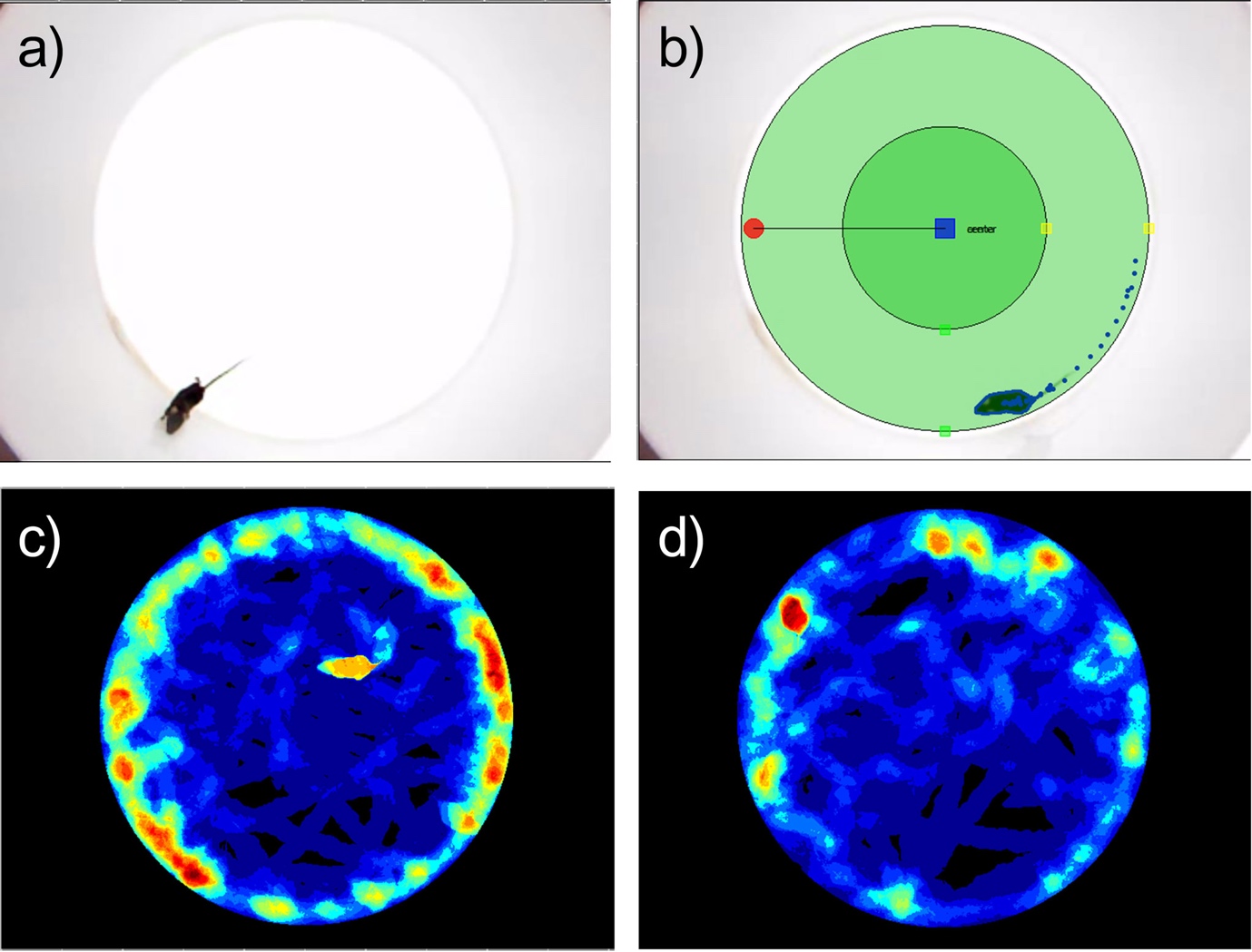


Supplementary Figure S1. Open field test: a) picture of the arena from the overhead digital camera, b) the zonal division of the arena, c) representative density map for the wild-type mouse, d) representative density map for the *Ptpn5* KO strain. The density maps show location of animal-associated pixels in the arena throughout the test session (A. V. Kulikov et al., 2008).


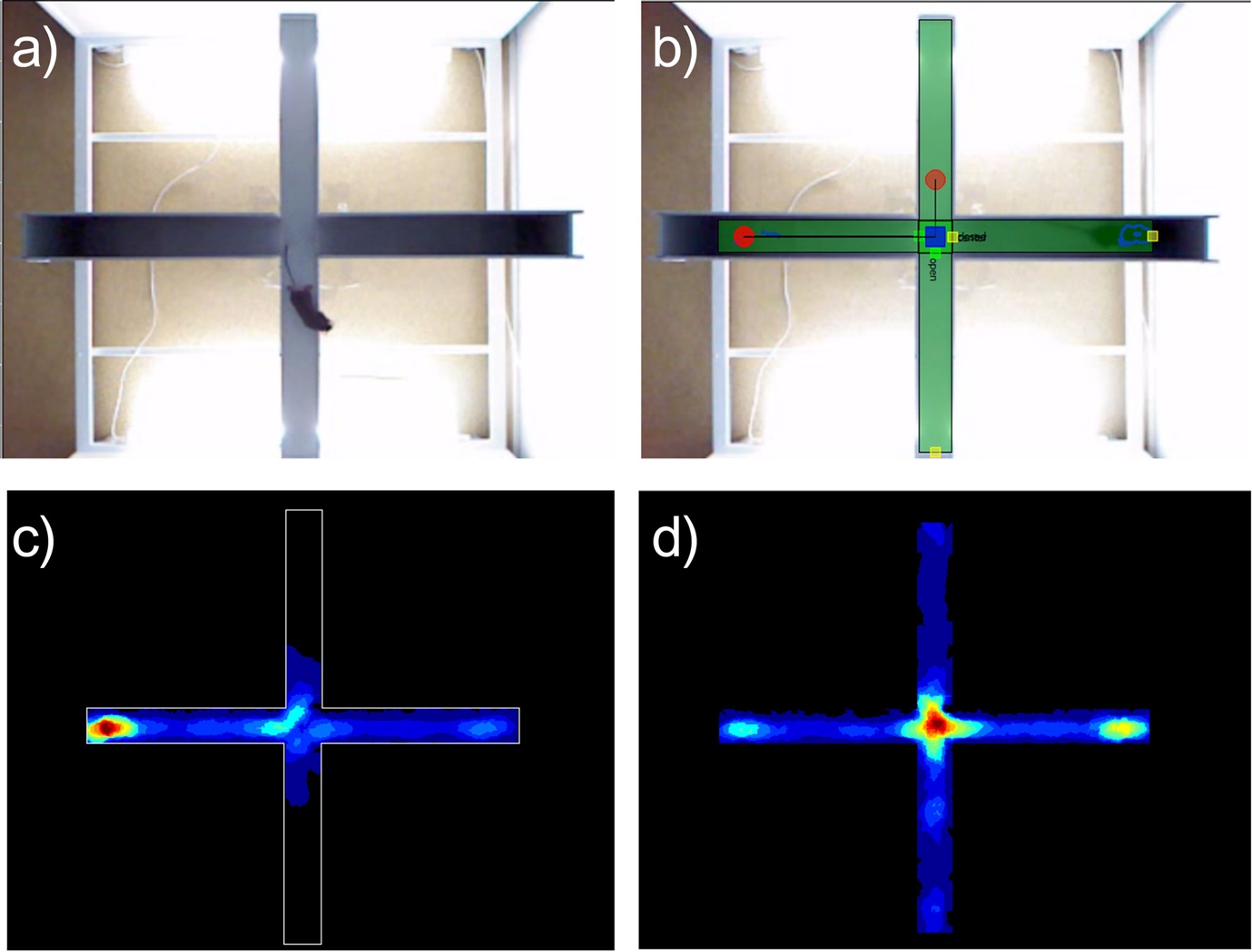


Supplementary Figure S2. Elevated plus maze test: a) picture of the arena from the overhead digital camera, b) the zonal division of the arena, c) representative density map for the wild-type mouse, d) representative density map for the *Ptpn5* KO strain. The density maps show location of animal-associated pixels in the arena throughout the test session (A. V. Kulikov et al., 2008).


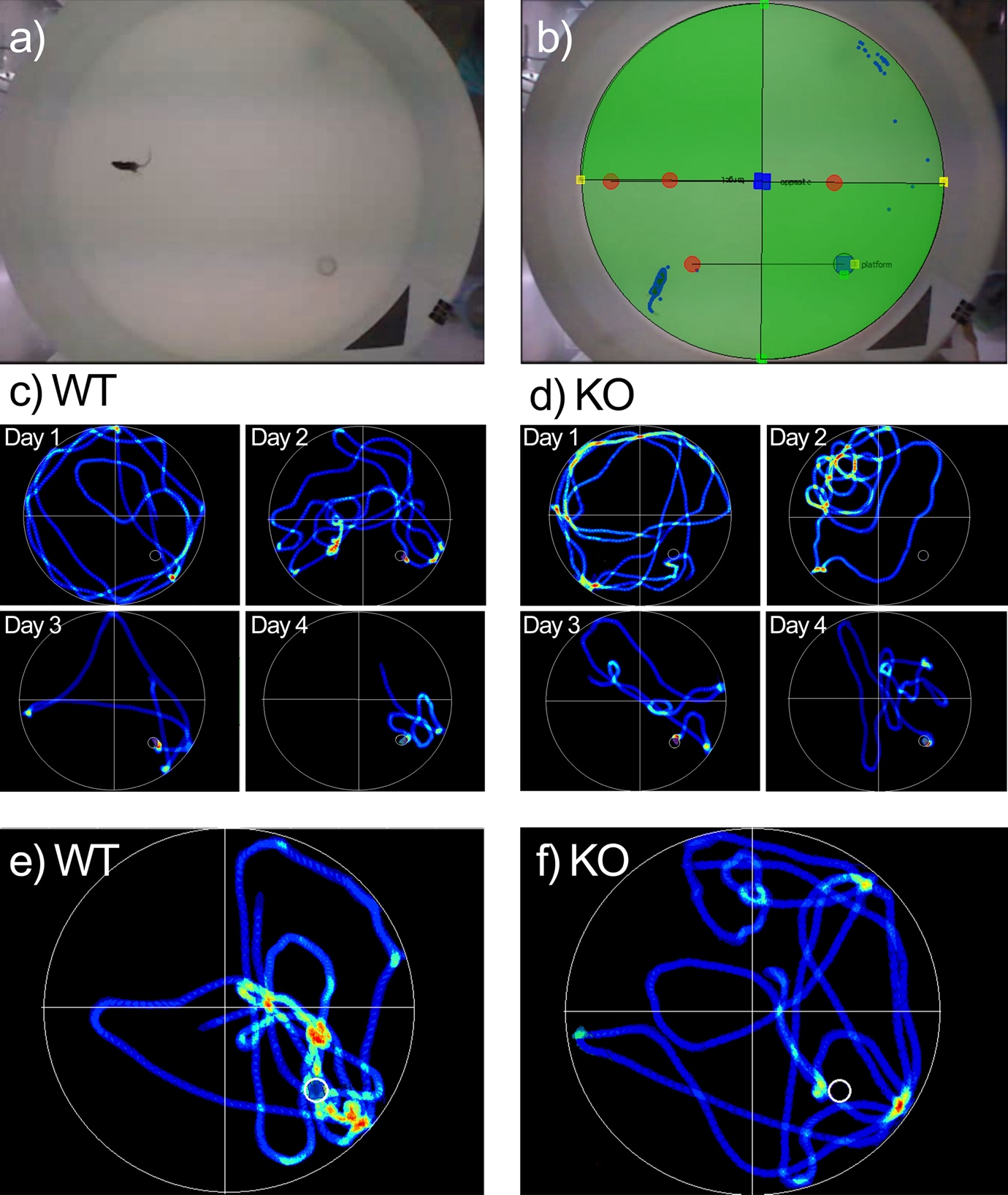


Supplementary Figure S3. Morris water maze test: a) picture of the arena from the overhead digital camera, b) the sectoral division of the arena, c) representative density map of the learning phase of the test (days 1-4) for the wild-type mouse, d) representative density map of the learning phase of the test (days 1-4) for the *Ptpn5* KO strain, e) representative density map of the memory retention phase of the test (day 5) for the wild-type mouse, f) representative density map of the memory retention phase of the test (day 5) for the Ptpn5 KO strain. The density maps show location of animal-associated pixels in the arena throughout the test session (A. V. Kulikov et al., 2008).


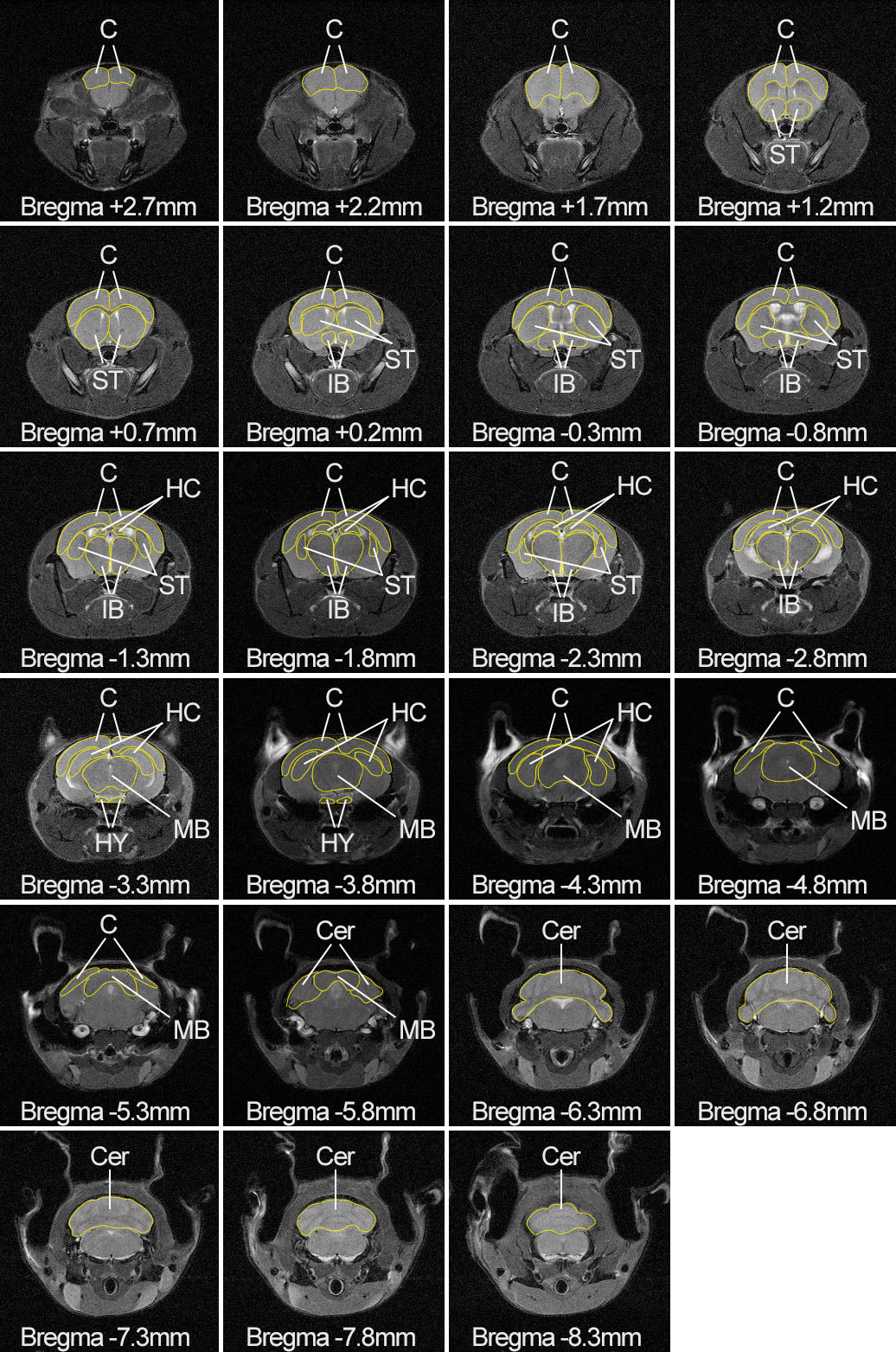


Supplementary Figure S4. Representative MRI slices with restricted brain regions used for the regions’ volume calculation. C – cortex, Cer – cerebellum, HC – hippocampus, HY – pituitary, IB – interbrain, M – midbrain, ST – striatum. Volumes of the brain structures and total volume of the brain were estimated using 23 slices of coronal orientation (slice thickness: 0.5 mm, inter-slice gap: 0 mm) and calculated as a sum of the areas of slices multiplied by 0.5 mm. The areas of structures in each slice were calculated as the number of pixels (measured with the ImageJ software) multiplied by the size of one pixel in mm^2^.

Supplementary Table S1. Results and statistical values obtained in behavioral tests (6-8 animals in group). Data are presented as mean ± SD. Groups were compared with T-test for independent samples for normal distribution and with Mann-Whitney U-test otherwise. Statistically significant differences are highlighted in bold.

| Behavioral parameter | Wild type | *Ptpn5* KO | Statistical values |
| --- | --- | --- | --- |
| ***Home cage behavior, averaged through 2 days*** | | | |
| Locomotor activity (m/h) | 38.41 ± 3.26 | 47.42 ± 9.34 | t_14_ = 2.58, ***p < 0.05** |
| Sleep duration (min/h) | 17.78 ± 0.76 | 15.68 ± 1.27 | t_14_ = 4.01, ****p < 0.01** |
| Food consumption (g/2h) | 0.33 ± 0.06 | 0.36 ± 0.06 | t_14_ = 0.89, p > 0.05 |
| Water consumption (g/2h) | 0.37 ± 0.07 | 0.44 ± 0.16 | t_14_ = 1.06, p > 0.05 |
| ***Operant wall*** |  |  |  |
| Day 2, number of nose pokes | 229.75 ± 131.72 | 186 ± 114.91 | t_13_ = 0.68, p > 0.05 |
| Day 2, number of pellets | 9 ± 1.93 | 6.75 ± 4.65 | U = 25, p > 0.05 |
| Day 3, number of nose pokes | 110.75 ± 49.85 | 154.71 ± 104.23 | t_13_ = 1.07, p > 0.05 |
| Day 3, number of pellets | 6.13 ± 2.64 | 6.38 ± 4.1 | U = 29, p > 0.05 |
| ***Open field*** | | | |
| distance traveled, m | 33.83 ± 7.21 | 40.92 ± 11.37 | t_13_ = 1.46, p > 0.05 |
| time spent in the center of the arena, % | 9.44 ± 2.83 | 10.51 ± 2.75 | t_13_ = 0.74, p > 0.05 |
| duration of rearing, s | 22.25 ± 6.11 | 21.47 ± 4.93 | t_13_ = 0.79, p > 0.05 |
| duration of grooming, s | 0.58 ± 1.27 | 3.33 ± 2.03 | U = 9, ***p < 0.05** |
| ***Social interaction test*** | | | |
| duration of social contacts, s | 116.74 ± 54.38 | 134.96 ± 46.31 | t_13_ = 0.70, p > 0.05 |
| ***Rotarod test*** | | | |
| latency to fall from the rod, s | 152.96 ± 51.56 | 134.75 ± 54.47 | t_14_ = 0.69, p > 0.05 |
| ***Three-chambered test*** | | | |
| time spent in the chamber with mouse, % | 69.75 ± 7.73 | 73.11 ± 7.32 | t_13_ = 0.86, p > 0.05 |
| ***Novel object recognition test*** | | | |
| time spent near the novel object, % | 60.23 ± 17.3 | 62.78 ± 17.65 | t_14_ = 0.29, p > 0.05 |
| ***Elevated plus maze test*** | | | |
| duration of stretch postures, sec | 43.26 ± 11.75 | 49.39 ± 7.71 | t_13_ = 1.17, p > 0.05 |
| distance traveled, m | 13.84 ± 1.24 | 14.33 ± 0.92 | t_13_ = 0.86, p > 0.05 |

Supplementary Table S2. Effects of the *Ptpn5* gene functional knockout on serotonin metabolism index (5-HT/5-HIAA) in the brain of mice (7-8 animals in group). Data are presented as mean ± SD. Groups were compared with T-test for independent samples.

| Brain structure | Wild type | *Ptpn5* KO | Statistical values |
| --- | --- | --- | --- |
| Hippocampus | 0.66 ± 0.13 | 0.75 ± 0.04 | t_12_ = 1.49, p > 0.05 |
| Frontal cortex | 0.35 ± 0.08 | 0.35 ± 0.08 | t_13_ = 0.04, p > 0.05 |
| Striatum | 0.56 ± 0.03 | 0.62 ± 0.09 | t_12_ = 1.61, p > 0.05 |
| Midbrain | 0.91 ± 0.13 | 0.89 ± 0.13 | t_13_ = 0.32, p > 0.05 |

Supplementary Table S3. Effects of the *Ptpn5* gene functional knockout on the tryptophan hydroxylase 2 activity and expression in the brain of mice. Enzymatic activity was calculated as pmol of 5-hydroxytryptofan produced in 1 min per 1 mg of protein in the probe. The gene expression level was evaluated as the number of transcript copies per 100 copies of *Polr2a* mRNA (7-8 animals in group). Data are presented as mean ± SD. Groups were compared with T-test for independent samples.

|  | Wild type | *Ptpn5* KO | Statistical values |
| --- | --- | --- | --- |
| TPH2 activity, pmol/min per 1 mg of protein | | | |
| Hippocampus | 2.41 ± 0.62 | 2.31 ± 0.57 | t_14_ = 0.35, p > 0.05 |
| Frontal cortex | 2.86 ± 0.42 | 2.51 ± 0.6 | t_14_ = 1.37, p > 0.05 |
| Striatum | 0.51 ± 0.05 | 0.46 ± 0.11 | t_13_ = 1.10, p > 0.05 |
| Midbrain | 58.48 ± 13.71 | 63.82 ± 23.26 | t_13_ = 0.53, p > 0.05 |
| *Tph2* gene expression | | | |
| Midbrain | 87.50 ± 42.77 | 68.09 ± 50.41 | t_14_ = 0.83, p > 0.05 |

Supplementary Table S4. Effects of the *Ptpn5* gene functional knockout on the monoamine oxidase A expression in the brain of mice. The gene expression levels were evaluated as the number of transcript copies per 100 copies of *Polr2a* mRNA. The protein levels are presented as a percentage of the GAPDH protein level (6-8 animals in group). Data are presented as mean ± SD. Groups were compared with T-test for independent samples.

| Brain structure | Wild type | *Ptpn5* KO | Statistical values |
| --- | --- | --- | --- |
| *Maoa* gene expression | | | |
| Hippocampus | 48.76 ± 6.08 | 47.49 ± 3.77 | t_14_ = 0.50, p > 0.05 |
| Frontal cortex | 288.14 ± 33.24 | 296.58 ± 58.83 | t_13_ = 0.35, p > 0.05 |
| Striatum | 114.90 ± 15.59 | 117.89 ± 15.58 | t_13_ = 0.37, p > 0.05 |
| Midbrain | 573.62 ± 44.14 | 608.79 ± 50.72 | t_11_ = 1.38, p > 0.05 |
| MAOA protein level | | | |
| Hippocampus | 75.21 ± 21.89 | 80.76 ± 7.03 | t_13_ = 0.64, p > 0.05 |
| Frontal cortex | 140.29 ± 10.98 | 124.84 ± 53.42 | t_12_ = 0.69, p > 0.05 |
| Striatum | 87.47 ± 14.85 | 94.24 ± 20.44 | t_13_ = 0.72, p > 0.05 |
| Midbrain | 70.59 ± 13.53 | 68.19 ± 10.48 | t_14_ = 0.40, p > 0.05 |

Supplementary Table S5. Effects of the *Ptpn5* gene knockout on the serotonin transporter expression in the brain of mice. The gene expression levels were evaluated as the number of transcript copies per 100 copies of *Polr2a* mRNA. The protein levels are presented as a percentage of the GAPDH protein level (7-8 animals in group). Data are presented as mean ± SD. Groups were compared with T-test for independent samples.

| Brain structure | Wild type | *Ptpn5* KO | Statistical values |
| --- | --- | --- | --- |
| *Slc6a4* gene expression | | | |
| Midbrain | 36.02 ± 17.39 | 28.47 ± 23.36 | t_14_ = 0.73, p > 0.05 |
| 5-HTT protein level | | | |
| Hippocampus | 90.06 ± 28.62 | 77.61 ± 40.33 | t_13_ = 0.68, p > 0.05 |
| Frontal cortex | 170.86 ± 25.15 | 171.10 ± 36.72 | t_14_ = 0.02, p > 0.05 |
| Striatum | 66.09 ± 15.75 | 69.37 ± 14.21 | t_14_ = 0.44, p > 0.05 |
| Midbrain | 139.27 ± 32.51 | 131.03 ± 21.27 | t_14_ = 0.60, p > 0.05 |

Supplementary Table S6. Effects of the *Ptpn5* gene knockout on 5-HT_1A_ receptor expression and functional activity in the brain of mice. Receptor functional activity was measured as the body temperature change in response to administration of 5-HT_1A_ agonist 8-OH-DPAT (1 mg/kg, i.p.). The protein levels are presented as a percentage of the GAPDH protein level (6-8 animals in group). Data are presented as mean ± SD. Groups were compared with T-test for independent samples.

|  | Wild type | *Ptpn5* KO | Statistical values |
| --- | --- | --- | --- |
| 5-HT_1A_ functional activity | | | |
| Body temperature change | 2.34 ± 0.91 | 2.23 ± 0.67 | t_14_ = 0.26, p > 0.05 |
| 5-HT_1A_ protein level | | | |
| Hippocampus | 91.53 ± 10.61 | 97.09 ± 31.21 | t_13_ = 0.45, p > 0.05 |
| Frontal cortex | 77.31 ± 20.07 | 80.97 ± 30.01 | t_13_ = 0.28, p > 0.05 |
| Striatum | 50.94 ± 15.27 | 60.86 ± 6.32 | t_12_ = 1.49, p > 0.05 |
| Midbrain | 48.63 ± 3.59 | 51.36 ± 13.14 | t_13_ = 0.53, p > 0.05 |

Supplementary Table S7. Effects of the *Ptpn5* gene knockout on 5-HT_2A_ receptor expression and functional activity in the brain of mice. Receptor functional activity was measured as number of head-twitches in 20 min after administration of 5-HT_2A_ agonist DOI (1 mg/kg, i.p.). The gene expression levels were evaluated as the number of transcript copies per 100 copies of *Polr2a* mRNA. The protein levels are presented as a percentage of the GAPDH protein level (6-8 animals in group). Data are presented as mean ± SD. Groups were compared with T-test for independent samples.

|  | Wild type | *Ptpn5* KO | Statistical values |
| --- | --- | --- | --- |
| 5-HT_2A_ functional activity | | | |
| Head twitches | 23.5 ± 3.85 | 20.75 ± 8.1 | t_14_ = 0.87, p > 0.05 |
| *Htr2a* gene expression | | | |
| Hippocampus | 5.72 ± 0.66 | 5.87 ± 0.36 | t_12_ = 0.51, p > 0.05 |
| Frontal cortex | 97.32 ± 16.75 | 114.77 ± 34.2 | t_13_ = 1.28, p > 0.05 |
| Striatum | 38.69 ± 6.41 | 46.57 ± 13.16 | t_11_ = 1.33, p > 0.05 |
| Midbrain | 18.71 ± 4.22 | 18.73 ± 5.31 | t_13_ = 0.01, p > 0.05 |
| 5-HT_2A_ protein level | | | |
| Hippocampus | 123.88 ± 16.71 | 128.65 ± 14.21 | t_13_ = 0.59, p > 0.05 |
| Frontal cortex | 164.22 ± 41.18 | 175.91 ± 29.73 | t_14_ = 0.65, p > 0.05 |
| Striatum | 261.98 ± 53.28 | 230.19 ± 45.43 | t_14_ = 1.28, p > 0.05 |
| Midbrain | 152.47 ± 35.73 | 139.69 ± 21.12 | t_14_ = 0.87, p > 0.05 |

Supplementary Table S8. Effects of the *Ptpn5* gene knockout on 5-HT_7_ receptor expression and functional activity in the brain of mice. Receptor functional activity was measured as the body temperature change in response to administration of selective 5-HT_7_ receptor agonist LP44 (4-[2-(methylthio)phenyl]-N-(1,2,3,4-tetrahydro-1-naphthalenyl)-1-piperazinehexanamide hydrochloride) (20.5 nM, i.c.v.). The protein levels are presented as a percentage of the GAPDH protein level (6-8 animals in group). Data are presented as mean ± SD. Groups were compared with T-test for independent samples.

|  | Wild type | *Ptpn5* KO | Statistical values |
| --- | --- | --- | --- |
| 5-HT_7_ functional activity | | | |
| Body temperature change | 1.84 ± 0.8 | 1.83 ± 0.99 | t_13_ = 0.01, p > 0.05 |
| 5-HT_7_ protein level | | | |
| Hippocampus | 111.48 ± 34.14 | 92.88 ± 20.13 | t_14_ = 1.33, p > 0.05 |
| Frontal cortex | 86.29 ± 33.95 | 73.02 ± 20.42 | t_14_ = 0.95, p > 0.05 |
| Striatum | 163.64 ± 21.37 | 175.85 ± 25.47 | t_14_ = 1.04, p > 0.05 |
| Midbrain | 121.53 ± 40.52 | 125.76 ± 53.44 | t_14_ = 1.78, p > 0.05 |
